# The Interplay of M1 Macrophages and Dental Pulp Stem Cells Promotes Angiogenesis Through IL‐8‐Dependent VEGF Regulation: An In Vitro Study

**DOI:** 10.1111/iej.70198

**Published:** 2026-06-24

**Authors:** Dineshi Sewvandi Thalakiriyawa, Mohamad Koohi‐Moghadam, Mingxin Hu, Hong Wang, Colman McGrath, Waruna Lakmal Dissanayaka

**Affiliations:** ^1^ Applied Oral Sciences & Community Dental Care, Faculty of Dentistry The University of Hong Kong Hong Kong SAR China; ^2^ Department of Diagnostic Radiology, Li K Shing Faculty of Medicine The University of Hong Kong Hong Kong SAR China; ^3^ Restorative Dental Sciences, Faculty of Dentistry The University of Hong Kong Hong Kong SAR China

**Keywords:** Angiogenesis, Dental pulp stem cells, IL‐8, Macrophages, Vascular endothelial growth factor

## Abstract

**Background:**

Dental pulp inflammation triggers immune responses involving macrophages and dental pulp stem cells (DPSCs), which interact to regulate angiogenesis essential for tissue repair. M1 pro‐inflammatory macrophages predominate early in pulpitis, and clarifying their angiogenic role is vital in identifying inflammatory regenerative mechanisms.

**Methodology:**

THP‐1 cells and peripheral blood monocyte (PBM)‐derived macrophages were polarized to M1 or M2 phenotypes, characterized by qRT‐PCR, ELISA, and angiogenesis arrays. A vasculature‐on‐a‐chip comprising DPSCs, human umbilical vein endothelial cells (HUVECs) and THP‐1‐derived macrophages was imaged, and the vascular segments/sprouts were quantified using ImageJ. Density effects used 5 × 10^4^ versus 7.5 × 10^4^ M1 macrophages/device, with propidium iodide staining for cytotoxicity. IL‐8 effects on DPSC VEGF secretion were assessed by ELISA (with/without Reparaxin 1 μM), Matrigel tube formation assays, and exogenous IL‐8 (0.5 ng/mL). Transwell co‐cultures underwent RNA sequencing and bioinformatics analysis, which identified candidate hub genes and signalling pathways; the results were validated by Western blotting (p‐ERK, HIF‐1α; ERK inhibitor SCH772984, 25 nM). Statistical testing was performed using ANOVA with Tukey's post hoc test (*p* < 0.05).

**Results:**

M1 macrophages at low density (5 × 10^4^ cells/device) significantly enhanced vascularization in the vasculature‐on‐a‐chip, increasing vascular segments (*p* < 0.0001) and free sprouts (*p* < 0.05–0.01) compared to M0 or no‐macrophage controls, with effects comparable to M2. High‐density M1 seeding (7.5 × 10^4^ cells/device) reduced sprouts (*p* < 0.0001 day 4, *p* < 0.01 day 5) due to increased cytotoxicity (*p* < 0.0001). Both THP‐1‐ and PBM‐derived M1‐conditioned media (CM) showed significantly elevated IL‐8 levels. M1 CM (THP‐1/PBM) induced DPSC VEGF secretion, blocked by Reparaxin (*p* < 0.01–0.0001), confirming IL‐8 mediation via CXCR1/2. Exogenous IL‐8 (0.5 ng/mL) upregulated DPSC VEGF protein/mRNA (*p* < 0.05) and Matrigel tube formation (segments/junctions *p* < 0.05). M1‐DPSC CM enhanced vascular meshes/segments on Matrigel (*p* < 0.05), reduced by Reparaxin. RNA‐seq of M1 co‐cultured DPSCs identified 17 angiogenic genes (logFC > 1.2), with HIF‐1α as a hub gene and an enriched MAPK/ERK pathway. Western blot analysis confirmed MAPK/ERK‐HIF‐1α as a contributory pathway in IL‐8‐induced upregulation of VEGF in DPSCs.

**Conclusion:**

M1 macrophages promote angiogenesis via IL‐8‐induced DPSC VEGF secretion through CXCR1/2‐MAPK/ERK‐HIF‐1α signalling density‐dependently, suggesting that therapeutic modulation rather than total suppression of M1 activity could improve outcomes in vital pulp therapy.

## 1 Introduction

Dental pulp tissue, in response to bacterial invasion and by‐products, initiates a robust immunoinflammatory response (Duncan and Cooper 2020; Farges et al. 2015; Ji et al. 2022). Host cells detect carious infection and trigger this defence by secreting cytokines (such as IL‐1, IL‐6 and IL‐8) to recruit neutrophils and macrophages (McLachlan et al. 2005, 2004). This process aims to contain and eliminate the infection while preparing the tissue for repair. Central to both healing and inflammation is the vasculature; deep caries exhibit an increased blood vessel density to deliver immune cells and clear harmful toxins or metabolites before reparative dentine forms. Consequently, macrophages, perivascular cells, dental pulp stem cells (DPSCs) and endothelial cells must interact closely within this microenvironment (Eming et al. 2014; Szade et al. 2015).

Macrophages are key regulators of this pulpal response and serve as vital histological indicators of the stages of pulpitis (Contreras et al. 2023; Guo et al. 2024). Distinct from other immune cells, they participate in inflammation while remaining critical to tissue homeostasis, regeneration and repair (Huang et al. 2023). Human and rodent pulpitis models show a clear spatial distribution: pro‐inflammatory M1 macrophages gather in high numbers near the lesion or exposure site, whereas pro‐regenerative M2 macrophages reside further away in the deeper pulp but migrate near the lesion during successful pulp capping to promote repair (Contreras et al. 2023; Huang et al. 2023; Yoshiba et al. 2020). Therefore, the evidence suggests that M1 macrophages dominate active inflammation (Beyer et al. 2018) and may exert anti‐regenerative effects (Jetten et al. 2014), while M2 phenotypes drive regeneration.

More recently, Graney et al. provided nuanced insights into these dynamics. They showed that short‐term exposure (1 day) of adipose‐derived mesenchymal stem cells and endothelial cells to M1 macrophages increased vessel formation, whereas longer exposure (3 days) caused vessel regression. This reveals that the angiogenic effects of M1 macrophages are highly dependent on timing, exposure duration and specific microenvironmental conditions. The evidence indicates that M1 macrophages have a more complex role in angiogenesis than previously thought, challenging simple pro‐ or anti‐angiogenic classifications (Graney et al. 2020).

DPSCs also support repair through their potent angiogenic and immunomodulatory properties. When exposed to inflammatory stimuli, DPSCs elicit an angiogenic response by secreting exosomes and other extracellular vesicles containing pro‐angiogenic miRNAs (Zhou et al. 2021, 2020). For instance, short‐term exposure to tumour necrosis factor‐alpha (TNF‐α) upregulates their expression of vascular endothelial growth factor (VEGF) (Boyle et al. 2014), a predominant factor that induces endothelial cell proliferation, sprouting and tube formation (Dissanayaka et al. 2015; Hilkens et al. 2017). Indeed, DPSCs are recognized as an effective source of VEGF (Bronckaers et al. 2013; Dasgupta et al. 2023; Dissanayaka et al. 2015; Grando Mattuella et al. 2007; Han et al. 2020). Moreover, their immunomodulatory role allows them to mitigate inflammation and guide macrophage polarization into the pro‐healing M2 phenotype (Di Tinco et al. 2021; Gong et al. 2026; Ma et al. 2025).

Taken together, this evidence suggests that both M1 macrophages and DPSCs may mediate the initial vascular response in pulp inflammation. However, how M1 macrophages directly influence the angiogenic properties of DPSCs remains unknown. This study aimed to investigate the novel interaction between M1 macrophages and DPSCs in mediating angiogenesis, focusing on the specific cytokines, growth factors and intracellular signalling pathways activated in DPSCs. The findings of the study will expand our understanding of cellular crosstalk during pulp inflammation and repair and strengthen the evidence base for vital pulp therapy options.

## 2 Materials and Methods

### 2.1 Methodology

**FIGURE 1:**
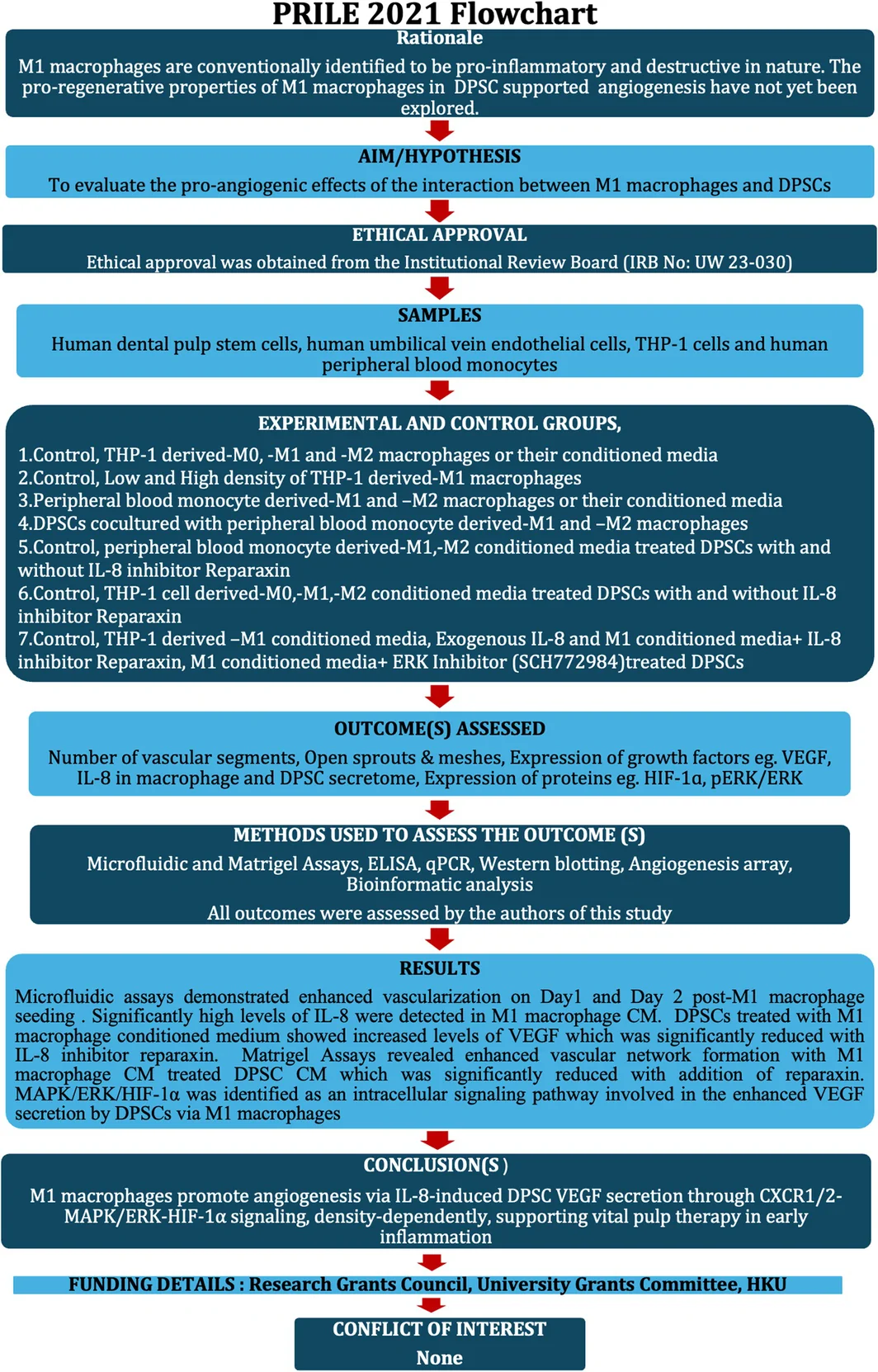
PRILE flow chart.

The manuscript of this laboratory study has been written according to the Preferred Reporting Items for Laboratory studies in Endodontology (PRILE) 2021 guidelines (Figure 1).

We adopted a vasculature‐on‐a‐chip microfluidic platform, which we previously reported, to establish a tri‐culture system comprising DPSCs, HUVECs and macrophages. This system aimed to recapitulate the pulp perivascular microenvironment and evaluate how different macrophage phenotypes influence DPSC‐supported vascular tube formation (Zhang et al. 2025, 2022). We primarily used THP‐1‐derived macrophages in functional and molecular assays, as they are the most established macrophages in laboratory studies. To validate the findings from THP‐1 cells, especially regarding the secretome and signalling pathways, we also employed peripheral blood monocyte (PBM)‐derived macrophages. This allowed us to compare cytokine profiles, considering that THP‐1 cells are not clinically applicable but do exhibit similarities and differences to PBM‐derived macrophages in cytokine secretion.

In our observations, tri‐cultures comprising THP‐1‐derived M1 macrophages at relatively low densities exhibited enhanced vascularization, similar to that observed in M2 cultures. To explore this, we analysed the secretomes of M1 and M2 macrophages derived from THP‐1 cells for angiogenic cytokines and growth factors. The analyses revealed high concentrations of IL‐8 in the M1 secretome and of VEGF in M2 macrophages.

Considering that VEGF is a pivotal angiogenic growth factor abundantly secreted by DPSCs, we investigated the impact of IL‐8 secreted by M1 macrophages on VEGF expression in DPSCs and on endothelial tube formation. These evaluations involved enzyme‐linked immunosorbent assay (ELISA) for VEGF quantification and a Matrigel assay to assess the effects of macrophage‐derived IL‐8 on initial steps of angiogenesis. Utilizing exogenous IL‐8 and the IL‐8 receptor inhibitor Reparaxin, we further substantiated the IL‐8‐mediated effects on the angiogenic properties of DPSCs.

Additionally, to elucidate the signalling pathways responsible for M1‐induced VEGF expression in DPSCs, we performed RNA sequencing coupled with bioinformatic analysis on DPSCs co‐cultured with PBM‐derived macrophages in a transwell system. These findings were corroborated through Western blot analysis.

### 2.2 Ethical Approval

Ethical approval for collecting peripheral blood to isolate monocytes from volunteers was obtained from the Institutional Review Board of the University of Hong Kong (IRB no: UW23‐030).

### 2.3 Cell Culture

DPSCs were purchased from Lonza (Basel, Switzerland) and cultured in α‐minimum essential medium (α‐MEM) supplemented with 10% fetal bovine serum (FBS) and 1% penicillin/streptomycin. The mesenchymal origin and multipotent differentiation capacity of the cells were evaluated and reported in our previous study (Zhang et al. 2021). HUVECs were purchased from ScienCell (Carlsbad, CA, USA) and cultured in endothelial cell medium (ECM, ScienCell) supplemented with 5% (v/v) FBS, 1% (v/v) endothelial cell growth supplement and 1% (v/v) penicillin/streptomycin. Green fluorescent protein (GFP)‐expressing human umbilical vein endothelial cells (GFP‐HUVECs) were purchased from Angio‐Proteomie (Boston, MA, USA) and cultured in ECM. All cell cultures were kept in a 37°C incubator with 5% CO_2_. Passage 5–8 of DPSCs, passage 4–8 of HUVECs and GFP‐HUVECs were used in all experiments.

### 2.4 Monocyte Cell Line

Tohoku Hospital Pediatrics‐1 (THP‐1) cells were a gift from Prof. Lijian Jin and were cultured in Rosswell Park Memorial Institute (RPMI) 1640 medium (ThermoFisher, Waltham, MA, USA) supplemented with L‐glutamine, 10% FBS and 1% penicillin/streptomycin.

**FIGURE 2:**
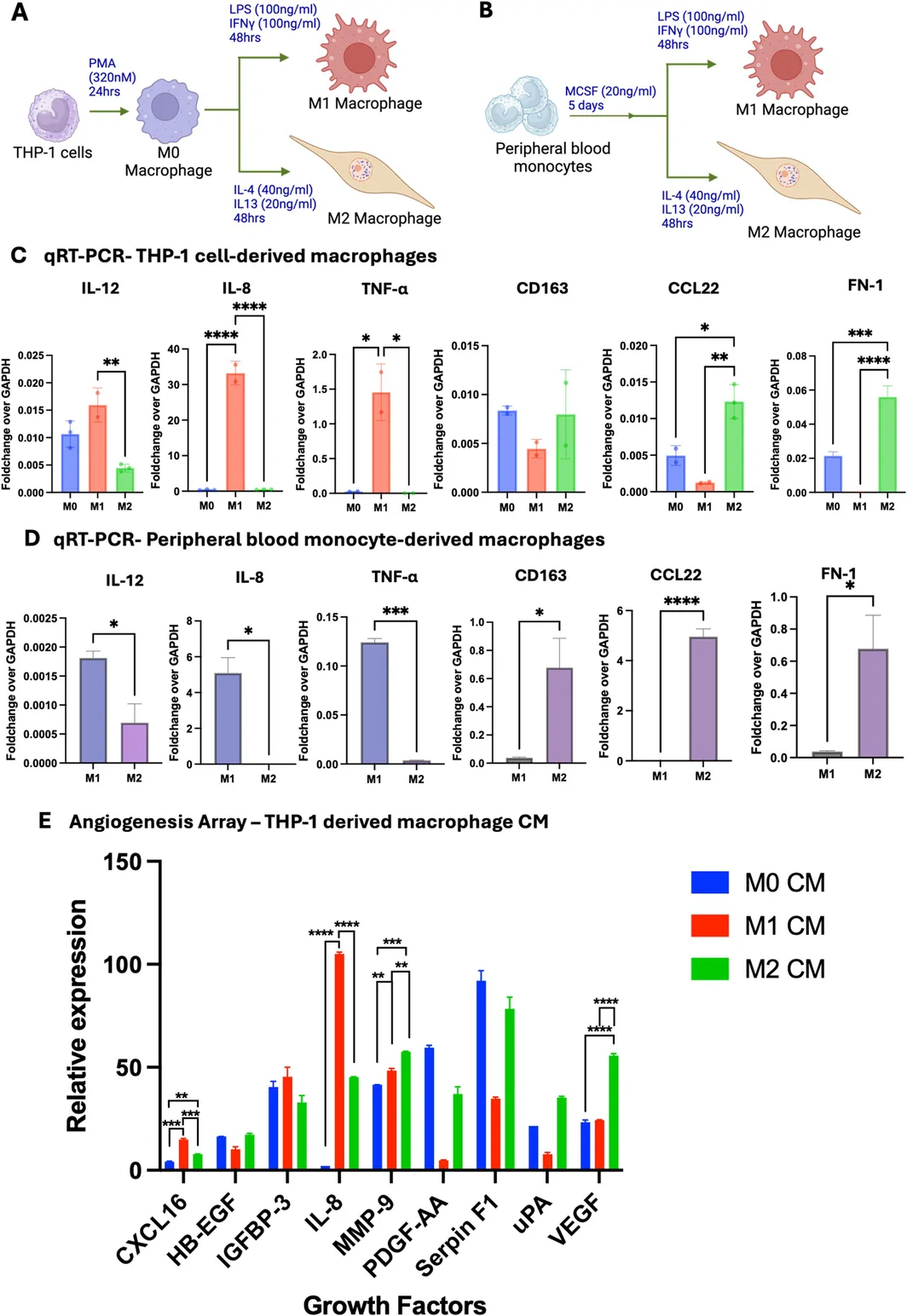
Cytokine expression profiles of THP‐1‐ and PBM‐derived macrophage phenotypes (A) Polarization of THP‐1‐derived macrophages. Created in BioRender. Dissanayaka, W. L. (2026) https://BioRender.com/9mu8aiz. (B) Polarization of PBM‐derived macrophages. Created in BioRender. Dissanayaka, W. L. (2026) https://BioRender.com/31yourh. (C–E) Characterization of macrophage phenotypes: mRNA expression of pro‐inflammatory genes: IL‐8, IL‐12, TNFα and anti‐inflammatory genes: CD163, CCL22, FN‐1 in (C) THP‐1 cell‐derived macrophages, (D) PBM‐derived macrophages; (E) Proteome profiler human angiogenesis array for THP‐1‐derived macrophage CM. *p < 0.05, **p < 0.01, ***p < 0.001, ****p < 0.0001.

THP‐1 cells were differentiated into M0 macrophages using Phorbol 12‐myristate 13‐acetate (PMA) (Sigma‐Aldrich, St. Louis, MO, USA) (320 nM) exposure for 24 h. M0 macrophages were then exposed to recombinant human IL‐4 (R&D Systems, Minneapolis, MN, USA) (40 ng/mL) and recombinant human IL‐13 (Thermofisher) (20 ng/mL) for 48 h for differentiation into M2 macrophages and lipopolysaccharide (LPS) (Sigma Aldrich, Saint Louis, USA) (100 ng/mL) and interferon (IFN)‐γ (R&D Systems, Minneapolis, USA) (100 ng/mL) for 48 h for differentiation into the M1 phenotype (Figure 2A).

Characterization of different macrophage phenotypes was done using the human angiogenesis array kit, quantitative real‐time polymerase chain reaction (qRT‐PCR) for pro‐ and anti‐inflammatory genes and ELISA for tumour necrosis factor (TNF)‐α (R&D Systems).

### 2.5 Primary Human Monocyte‐Derived Macrophages

Primary human monocytes were obtained from the peripheral blood of 4 healthy adult volunteers with informed consent. To minimize the influence of donor heterogeneity, strict inclusion criteria were applied; all donors were healthy, nonsmoking adults aged 25–35 years with no history of systemic inflammatory conditions. In addition, standard protocols were followed for macrophage differentiation, and phenotypic characterization confirmed consistent trends across replicates, supporting the reproducibility of the experimental outcomes. More importantly, monocytes of individual donors were pooled prior to usage in the experimental system, thereby reducing donor‐specific bias (Taciak et al. 2025). CD14+ monocytes were isolated using the Classical Monocyte Isolation Kit (Miltenyi Biotec, Germany) according to the manufacturer's instructions. Monocytes were cultured at a concentration of 1 × 10^5^ cells/well in 6‐well plates for 7 days in RPMI 1640 media, supplemented with 10% heat‐inactivated human serum, 1% penicillin/streptomycin and Macrophage colony‐stimulating factor (MCSF) (Sigma‐Aldrich) 20 ng/mL. Media were replenished on days 3 and 5. On day 5, the differentiated M0 macrophages were stimulated into the M1 and M2 phenotypes by the addition of IFN‐γ (100 ng/mL) and LPS (100 ng/mL) for M1 activation, IL‐4 (40 ng/mL) and IL‐13 (20 ng/mL) for M2 activation (Figure 2B). On day 7, all macrophages were washed with PBS to remove polarizing stimuli and used for downstream experiments.

Characterization of macrophage phenotypes was done using qRT‐PCR for pro‐ and anti‐inflammatory genes and ELISA for TNF‐α. PBM‐derived M0 macrophages were excluded from downstream assays because they exhibit complex transcriptional profiles that vary significantly with the duration of MCSF exposure, ranging from 5 to 9 days (Deng et al. 2022; Tarique et al. 2015).

### 2.6 RNA Extraction and Purification

Cell lysates were thawed on ice and mixed with equal volumes of 100% ethanol. The samples were then loaded into spin columns for purification according to the manufacturer's instructions (Accurate Biology. Hunan, China). The quantity and quality of RNA were measured using NanoDrop 1000 (Thermo Scientific).

Complementary DNA was synthesized from 1 μg of RNA using the cDNA Reverse Transcription Kit (Takara Bio. Shiga, Japan) (*A*_260/280_ > 1.8). qRT‐PCR was performed using Fast SYBR Green Master Mix (Takara) according to the manufacturer's instructions. Mean cycle threshold values were calculated from technical replicates. The expression of target genes was normalized to the reference gene glyceraldehyde‐3‐phosphate dehydrogenase (GAPDH). All primers were synthesized by Integrated DNA Technologies (IDT, Singapore):

CD163:5′‐TTTGTCAACTTGAGTCCCTTCAC‐3′ (forward) and 5′ TCCCGCTACACTTGT TTTCAC‐3′ (reverse), GAPDH:5′ AAGGTGAAGGTCGGAGTCAAC‐3′ (forward) and 5′ G GGGTCATTGATGGCAACAATA‐3′ (reverse). Fibronectin‐1: 5′ TGTGGTTGCCTTGCAC GAT‐3′ (forward) and 5′‐GCTTGTGGGTGTGACCTGAGT‐3′ (reverse) IL12: 5′‐AAAGGA CATCTGCGAGGAAAGTTC‐3′ (forward) and 5′‐CGAGGTGAGGTGCGTTTATGC 3′ (reverse), IL‐8: 5′‐TCTGTGTGAAGGTGCAGTTTT‐ 3′ (forward) and 5′‐GGGGTGGAAA GGTTTGGAGTA 3′ (reverse), TNFα: 5′‐CTGCACTTTGGAGTGATCGG‐3′ (forward) and 5′‐TCAGCTTGAGGGTTTGCTAC‐3′ (reverse), CCL22:5′TCATGGCTACCCTGCGTGTC‐3′, (forward) and 5′‐CCTTCACTAAACGTGGCAGAG‐3′ (reverse), VEGF: 5′‐GTACCTCC ACCATGCCAAGT‐3′ (forward) and 5′‐AATAGCTGCGCTGGTAGACG‐3′ (reverse).

### 2.7 Angiogenesis Array

To identify the array of angiogenic growth factors secreted by different macrophage phenotypes, Proteome Profiler Human Angiogenesis Array (R&D Systems) was conducted on THP‐1 cell‐derived macrophage conditioned medium (CM) according to the manufacturer's protocols. The experiment was conducted twice independently, with 2 technical replicates included in each trial.

### 2.8 ELISA

THP‐1 cell‐ and PBM‐derived macrophage CM were collected and analysed for VEGF, IL‐8 and TNF‐α using a commercially available ELISA Development Kit (R&D Systems, USA) according to the manufacturer's instructions.

Further, ELISA for VEGF was conducted on Macrophage CM‐treated DPSC supernatants with or without IL‐8 inhibitor Reparaxin (1 μM) (DF 1681Y) (MedChemexpress, NJ, USA), which exerts its effects via allosteric inhibition of IL‐8 receptors CXCR1 and CXCR2 (Souza et al. 2004). A 48‐h treatment period was chosen to allow detectable VEGF accumulation in the supernatant before the cells reached confluency, consistent with methodologies used in prior studies of IL‐8‐induced VEGF secretion in MSCs (Hou et al. 2014).

To confirm the IL‐8‐specific effects, DPSCs were treated with a range of concentrations of recombinant human IL‐8 (Sigma‐Aldrich) (0.1–5 ng/mL) for 48 h, and the concentration of VEGF in the supernatants was assessed using ELISA.

### 2.9 Microfluidic Chip Assay of Angiogenesis

**FIGURE 3:**
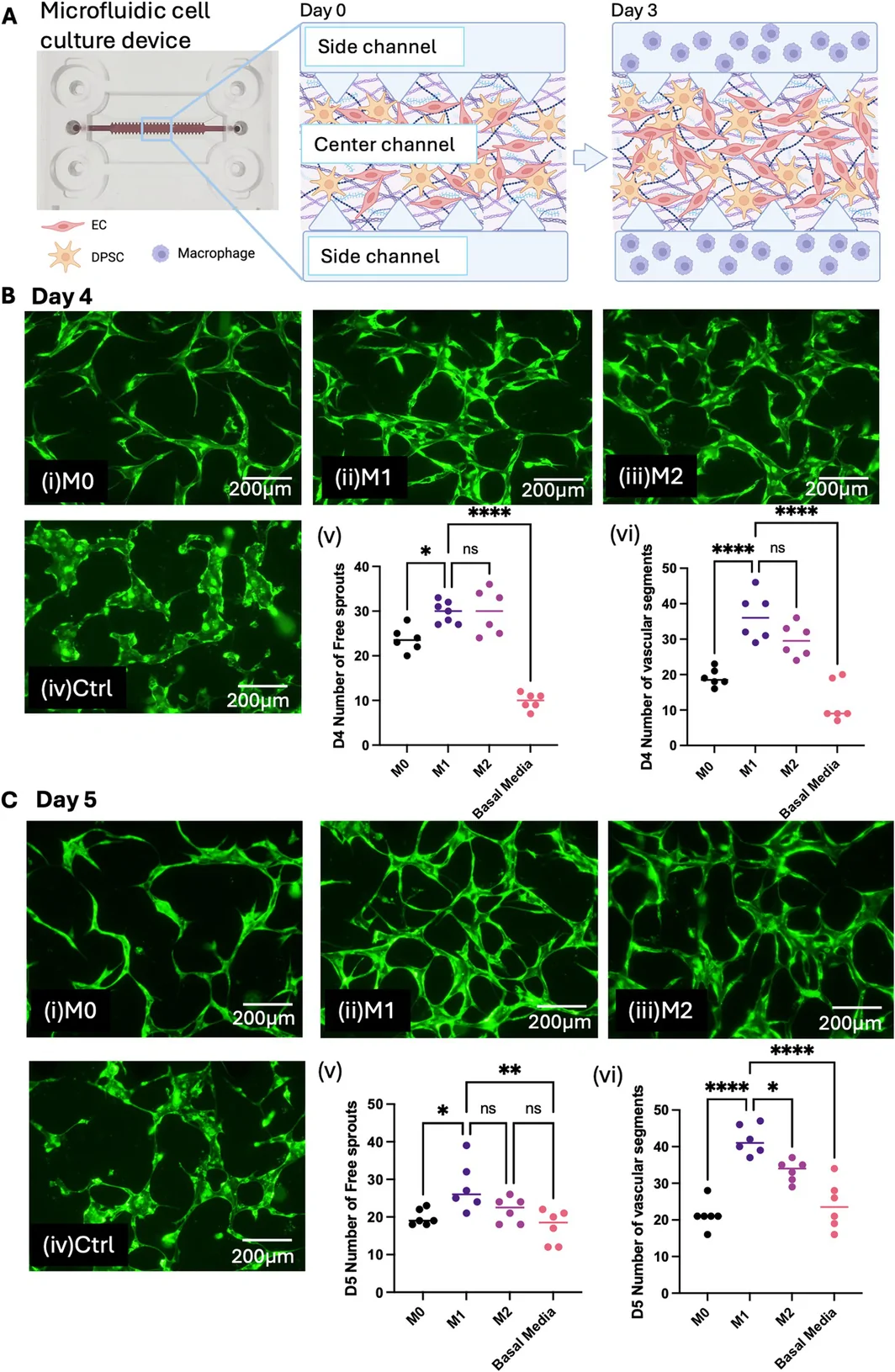
Effects of macrophage phenotypes on angiogenesis in a vasculature‐on‐a‐chip model. (A) DPSC‐GFP‐HUVEC‐macrophage and fibrin hydrogel configuration on the microfluidic device. Created in BioRender. Dissanayaka, W. L. (2026) https://BioRender.com/c8b7ah6. (B) Vascular sprouts on day 4, when (i) M0, (ii) M1, (iii) M2 and (iv) no macrophages (control) were added to the side channel; (v) Number of vascular segments; (vi) Number of free sprouts. (C) Vascular sprouts on day 5, when (i) M0, (ii) M1, (iii) M2, (iv) no macrophages (control) were added to the side channel; (v) Number of vascular segments; (vi) Number of free sprouts. * *p* < 0.05, **p < 0.01, ****p < 0.0001.

The interaction of macrophages with DPSC‐supported vascular constructs was recapitulated using the vasculature‐on‐a‐chip that we developed on a microfluidic device (AIM Biotech, Singapore), as indicated in Figure 3A (Zhang et al. 2025, 2022). This microfluidic device comprises three channels: a central channel and two side channels. DPSCs and GFP‐HUVECs (1:5) embedded in fibrin hydrogel (2.5 mg/mL) with Aprotinin (Sigma‐Aldrich) and thrombin (Sigma‐Aldrich) were loaded into the center channel of the microfluidic device. Co‐culture Media were added to the side channels and replenished daily for 2 days. THP‐1 cells were seeded in ultra‐low attachment well plates at a density of 1 × 10^6^ cells/well and differentiated into M0, M1 and M2 macrophages according to the protocol described previously. On day 3, differentiated THP‐1 cell‐derived M1, M2 and M0 macrophages were harvested using sterile cell scrapers and seeded in the side channels of microfluidic devices, at a concentration of 5 × 10^4^ cells per device. At this density, corresponding to a macrophage: DPSC ratio of 6.25:1, consistent with cell ratios employed in in vitro three‐dimensional models simulating inflammatory microenvironments (Romero‐López et al. 2020), reproducible and quantifiable changes in vascular parameters were observed, whereas lower macrophage densities failed to elicit such changes as observed in pilot studies. After seeding macrophages, tri‐culture media were added to the devices and replenished daily.

Fluorescent imaging of microfluidic devices was done daily using a Nikon TI‐SFL microscope. Three sites were imaged under 10× magnification in each microfluidic device, and the number of vascular segments and free sprouts in each image was quantified using ImageJ software. A total of three microfluidic devices were included per group.

To investigate whether the angiogenic effects of M1 macrophages were concentration‐dependent, M1 macrophages were seeded in two different densities (5 × 10^4^ cells/device and 7.5 × 10^4^ cells/device) on day 3 in the above setup and observed under fluorescence microscopy for 2 more days. On day 5, propidium iodide staining was used to assess the extent of cell death at each density, indicative of the cytotoxic effects induced by M1 macrophages.

The density of 7.5 × 10^4^ cells/device was selected to represent an escalated inflammatory state. Importantly, as revealed in our titration assays, densities exceeding this threshold led to widespread vascular regression and significant cell death. Therefore, the density of 7.5 × 10^4^ cells/device was employed to represent the maximum tolerable inflammatory load of the system, allowing us to study the transition toward severe inflammation without the confounding effects of total tissue collapse.

### 2.10 Matrigel Tube Formation Assay

To assess the effects of IL‐8 in Macrophage CM on vascularization, the Tube formation assay was conducted on Matrigel with Macrophage CM (24 h), treated DPSC supernatants with or without IL‐8 inhibitor Reparaxin (48 h) and exogenous IL‐8‐treated DPSC supernatants (48 h). 10 μL of Matrigel was added to 15‐well 3D μ slides—81 506 (Ibidi, Gräfelfing, Germany) and allowed to polymerize for 30 min at 37°C. Then, 1 × 10^4^ HUVECs, resuspended in respective supernatants, were seeded on top of Matrigel (Figure 5B). The dynamic progression of endothelial assembly was monitored at 4, 6, 8 and 12 h. Quantitative analysis was performed at the 8‐h time point, which was determined by preliminary kinetic studies to represent the peak of network complexity prior to the natural regression of the tubes. Three fields from each well at 10× magnification and one field from each well at 4× magnification were imaged under a Nikon DS Ri1 microscope. Tube formation, in terms of the number of vascular segments and meshes, was analysed using ImageJ, based on the average of three 4× images per condition. Experiments were performed three times independently.

### 2.11 In Vitro Transwell Co‐Culture of DPSCs and Macrophages

DPSCs (2 × 10^5^ cells/insert) were seeded in the apical chamber of Transwell inserts (0.4 μm pore size and 6.5‐mm diameter) in 1 mL of αMEM and cultured overnight at 37°C and 5% CO_2_. PBMs were seeded at a density of 1 × 10^5^ cells/well and differentiated into M1 and M2 macrophages following the previously mentioned protocol. Media were removed from all macrophage‐seeded wells and replaced with 2 mL of co‐culture media at a 1:1 ratio of αMEM to RPMI 1640 with MCSF to support cell survival. Transwell inserts containing DPSCs were transferred to macrophage‐seeded wells. All samples were incubated for 48 h at 37°C and 5% CO_2_. DPSCs cultured in the absence of macrophages in equal volumes of co‐culture media served as controls (Figure 6A).

After 48 h, the media was removed, and all samples were washed in 1X PBS to remove residual media. DPSCs were lysed directly in the inserts in 500 μL Trizol (Invitrogen, Carlsbad, CA, USA). All lysates were submitted for strand‐specific RNA sequencing. Three biological replicates were included per group.

### 2.12 RNA Extraction and Sequencing

The DPSC samples co‐cultured with M1 and M2 macrophages were harvested using Trizol and sent to BMK Gene Company (Beijing, China) for processing and analysis. The total RNA was extracted using the Tiangen DP762‐T1C kit (Tiangen Biotech, Beijing, China). The quantity and the purity of the isolated RNA were detected using Nanodrop2000 and Agilent2100, LabChip GX, respectively.

The total RNA samples were purified twice using mRNA capture beads (HieffNGS DNA selection Beads (Superior Ampure XPalternative) (Yeasen)), followed by fragmentation using a PCR instrument under specific conditions. This was followed by the synthesis of the first strand of complementary DNA (cDNA) using reverse transcription reagents and the second strand of cDNA using specific reagents for the first strand. Subsequently, adapter ligation was performed, after which the products were subjected to a cleanup procedure using magnetic beads and ethanol washes to remove impurities.

Library amplification was then carried out through PCR, followed by another clean‐up step to ensure high‐quality DNA samples. Finally, the library's quality was assessed using the Qsep‐400 system.

### 2.13 Bioinformatic Analysis

Raw data in fastq format were first processed using in‐house Perl scripts, yielding clean reads. All downstream analyses were based on high‐quality, clean data. Picard—tools v1.41 and samtools v0.1.18 were used to sort, remove duplicated reads and merge the BAM alignment results of each sample. GATK2 or Samtools software was used to perform SNP calling. Raw VCF files were filtered with the GATK standard filter method and other parameters.

StringTie was applied to assemble the mapped reads generated by Hisat2. An AS profile was employed to predict alternative splicing events in each sample and sort them into 12 types. Gene function was annotated based on Nr (NCBI non‐redundant protein sequences); Nt (NCBI non‐redundant nucleotide sequences); Pfam (Protein family); KOG/COG (Clusters of Orthologous Groups of proteins); Swiss‐Prot (A manually annotated and reviewed protein sequence database); KO (KEGG Ortholog database).

Differential expression analysis of two conditions/groups was performed using DESeq2 and analysed using edgeR. KOBAS software was used to test the statistical enrichment of differentially expressed genes in KEGG pathways. Fold Change ≥ 1.2 was set as the threshold for significantly differential expression.

The DEG sequences were blasted (BLASTX) against the genome of a related species (for which protein–protein interactions are available in the STRING database: http://string‐db.org/) to obtain predicted PPIs for these DEGs. The PPIs of these DEGs were visualized using the Centiscape v2.2 plugin in Cytoscape.

### 2.14 Western Blotting

Using RNA sequencing results, hypoxia‐inducible factor‐1α (HIF‐1α), a transcription factor linked to VEGF synthesis, was identified as a hub protein, and the MAPK and PI3K pathways were identified as significantly upregulated in DPSCs cocultured with M1 macrophages. An extensive literature search revealed that IL‐8 exerts its effects through the MAPK/ERK and PI3K/Akt pathways, and that HIF‐1α is a hub protein potentially involved in IL‐8‐induced VEGF secretion in DPSCs cocultured with M1 macrophages (Chan et al. 2017).

Even though the PI3K/Akt pathway is the most investigated in IL‐8‐induced downstream mechanisms (Yang et al. 2018), our preliminary investigations of P13K/Akt pathway enhancement in IL‐8 and M1 CM‐treated DPSCs yielded inconclusive results. Therefore, the second‐highest upregulated pathway according to our bioinformatics results, the MAPK/ERK pathway, which is also commonly implicated in IL‐8‐induced downstream effects, was investigated in this study.

To examine whether HIF‐1α and MAPK/ERK pathways are upregulated in response to IL‐8 in M1 CM, the following experimental procedure was followed. DPSCs were seeded on 6‐well plates at a density of 3 × 10^5^ cells per well. The following day, the wells were changed to reduced‐serum medium (αMEM with 0.2% FBS). After 24 h of serum starvation, M1 Macrophage CM (24 h) or 0.5 ng/mL IL‐8 were added to DPSCs and cultured for 3 h with or without Reparaxin and ERK inhibitor SCH772984. Total protein was collected using M‐PER protein extraction buffer containing 1× protease inhibitor cocktail (Vazyme, Nanjing, China). The total protein concentrations were quantified by a BCA kit (Vazyme) following vortexing and centrifugation at 14000 rpm for 10 min. A total amount of 10–20 μg of protein from each sample was subjected to 12% sodium dodecyl sulfate‐polyacrylamide gel electrophoresis (SDS–PAGE) followed by transfer onto the polyvinylidene difluoride (PVDF) membrane (Vazyme). The membranes were blocked for 1 h with 5% (w/v) BSA in 0.1% (v/v) Tween 20 (TBST) followed by overnight incubation at 4°C with corresponding primary antibodies, including mouse monoclonal HIF‐1α (1: 1000; Abcam), and mouse monoclonal anti αTubulin (1:1000 Abcam) or rabbit monoclonal anti‐p‐ERK42/44 (1:1000; Cell signalling Technology Danvers, MA, USA), followed by membrane stripping using stripping buffer (Abcam. Cambridge, England) and overnight incubation with rabbit monoclonal anti‐ERK42/44 (1:1000; Cell signalling), rabbit monoclonal anti‐GAPDH (1:1000; Cell Signaling Technology). After three 10‐min washes in TBST, the membranes were incubated with appropriate horseradish peroxidase‐conjugated secondary antibodies (Cell Signaling Technology) for 1 h at room temperature. After repeating the washing step, the target protein signal was measured by enhanced chemiluminescence (Westernbright Advansta Inc. San Jose, CA). Then, the membranes were visualized using a gel imaging system (Bio‐Rad, Hercules, CA). Protein expression was quantified using ImageJ. Three biological replicates were included per group.

### 2.15 Inhibition of ERK1/2 by SCH772984

ERK1/2 inhibitor SCH772984 was purchased from Medchemexpress (HY‐50846). A concentration of 25 nM was selected as the effective inhibitory concentration based on testing at a series of concentrations.

### 2.16 Statistical Analysis

All statistical analyses were conducted using Graphpad Prism software 10, using Student's *T*‐test and one‐way ANOVA with post hoc Tukey analysis. A *p*‐value of less than 0.05 was considered significant. Data are shown as Mean ± SEM.

## 3 Results

### 3.1 Characterization of Macrophage Phenotypes

Both THP‐1 cell‐derived and PBM‐derived macrophages showed distinctly increased markers for M1 and M2 phenotypes, as demonstrated by qRT‐PCR. In THP‐1 cell‐derived macrophages, mRNA expression of pro‐inflammatory cytokines, IL‐12 (*p* < 0.01), TNF‐α (*p* < 0.05) and IL‐8 (*p* < 0.0001) was significantly elevated in M1 macrophages, while anti‐inflammatory markers CCL22 (*p* < 0.05–0.01) and FN‐1 (*p* < 0.001–0.0001) were significantly increased in M2 macrophages (Figure 2C). Comparably, in PBM‐derived macrophages, mRNA expression of pro‐inflammatory cytokines, IL‐12 (*p* < 0.05), TNF‐α (*p* < 0.001) and IL‐8 (*p* < 0.05) were significantly elevated in M1 macrophages, while anti‐inflammatory markers CD163 (*p* < 0.05), CCL22 (*p* < 0.0001) and FN‐1 (*p* < 0.05) were significantly increased in M2 macrophages (Figure 2D).

The secretory profiles of THP‐1‐derived macrophages, assessed using the Angiogenesis Array kit, showed significantly increased pro‐inflammatory cytokines CXCL16 (*p* < 0.001) and IL8 (*p* < 0.0001) in M1 CM, and anti‐inflammatory or pro‐regenerative growth factors, VEGF (*p* < 0.0001), and MMP‐9 (*p* < 0.01) in M2 macrophage CM (Figure 2E). Further, pro‐inflammatory cytokine TNF‐α was detected in significantly elevated levels in both THP‐1 cell‐derived (*p* < 0.0001) and PBM‐derived (p < 0.001) M1 macrophages as shown by ELISA (Figure S1A).

### 3.2 M1 Macrophages Enhance Vascularization in DPSC‐HUVEC Constructs

A vasculature‐on‐a‐chip microfluidic system was utilized to investigate how macrophages of different phenotypes influence DPSC‐supported vascularization. DPSCs and GFP‐HUVECs encapsulated in fibrin hydrogel were injected into the center channel and M0, M1 or M2 macrophages were seeded in the side channels on Day 3 of the culture (Figure 3A). M1 macrophage‐seeded cultures displayed enhanced vascularization in terms of significantly increased number of vascular segments (*p* < 0.0001) and free sprouts (*p* < 0.05) on Day 4 compared to cultures without macrophages and cultures with M0 resting state macrophages. However, no significant difference in vascular parameters was observed between M1‐ and M2‐macrophage‐seeded cultures on Day 4 (Figure 3B).

On day 5, further enhanced vascularization was observed in M1‐seeded cultures, which displayed significantly higher numbers of vascular segments than M0 (*p* < 0.0001), M2 (*p* < 0.05) and control groups (*p* < 0.0001). The number of free sprouts was also significantly enhanced in M1‐seeded cultures on Day 5, compared to M0‐seeded cultures (*p* < 0.05) and cultures without macrophages (*p* < 0.01) (Figure 3C). The results suggested that M1 macrophages are pro‐angiogenic in short‐term cultures of up to day 5.

### 3.3 Vascularization in DPSC‐Supported Constructs Depends on the Density of Seeded M1 Macrophages

**FIGURE 4:**
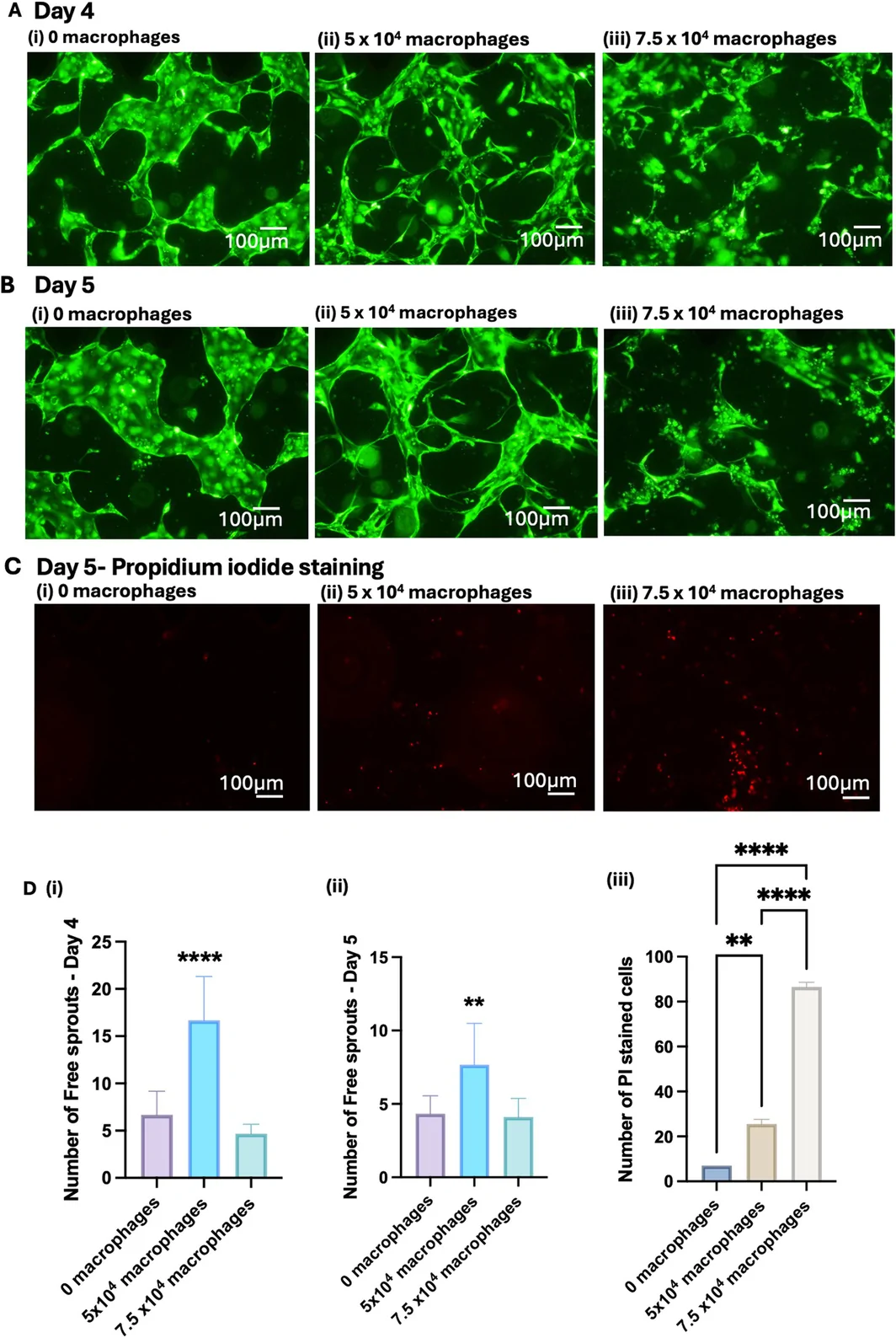
Density‐dependent effects of M1 macrophages on angiogenesis. Vascular sprouts on (A) day 4 and (B) day 5, when (i) 0 macrophages (control), (ii) macrophages at low density (5 × 10^4^macrophages) and (iii) macrophages at high density (7.5 × 10^4^macrophages) were seeded. (C) Propidium iodide staining on day 5 of cultures when (i) 0 macrophages (control), (ii) macrophages at low density (5 × 10^4^macrophages) and (iii) macrophages at high density (7.5 × 10^4^ macrophages) were seeded. (D) Average number of free sprouts on (i) Day 4, (ii) Day 5, (iii) Average number of PI‐stained cells on Day 5. **p < 0.01, ****p < 0.0001.

The same microfluidic experimental setup was subsequently used to investigate whether macrophage density determines their effects on the aforementioned angiogenic effects. M1 macrophages were seeded at two densities, and angiogenic effects were assessed for 5 days. On day 4 (day 1 post‐macrophage seeding) and day 5 (day 2 post‐macrophage seeding), the low‐density macrophage‐seeded cultures showed enhanced vascularization, quantified in terms of a significantly high number of free sprouts compared to the cultures with a higher density of macrophages and cultures with no macrophages (day 4‐*p* < 0.0001, day 5‐*p* < 0.01) (Figure 4A,B, (i) and (ii)). Propidium iodide staining was conducted to evaluate cell death across the three groups; higher‐density M1 constructs exhibited significantly more stained cells, indicating increased cytotoxicity (*p* < 0.0001) (Figure 4C,D (iii)). Therefore, the poor vascularization observed in the higher‐density cultures resulted from decreased cell survival. These findings imply that the pro‐angiogenic effects of M1 macrophages depend on density, with improved vascularization observed only at relatively low macrophage densities.

### 3.4 IL‐8 Secreted by M1 Macrophages Contributed to Endothelial Vascular Tube Formation by Inducing DPSC VEGF Secretion

**FIGURE 5:**
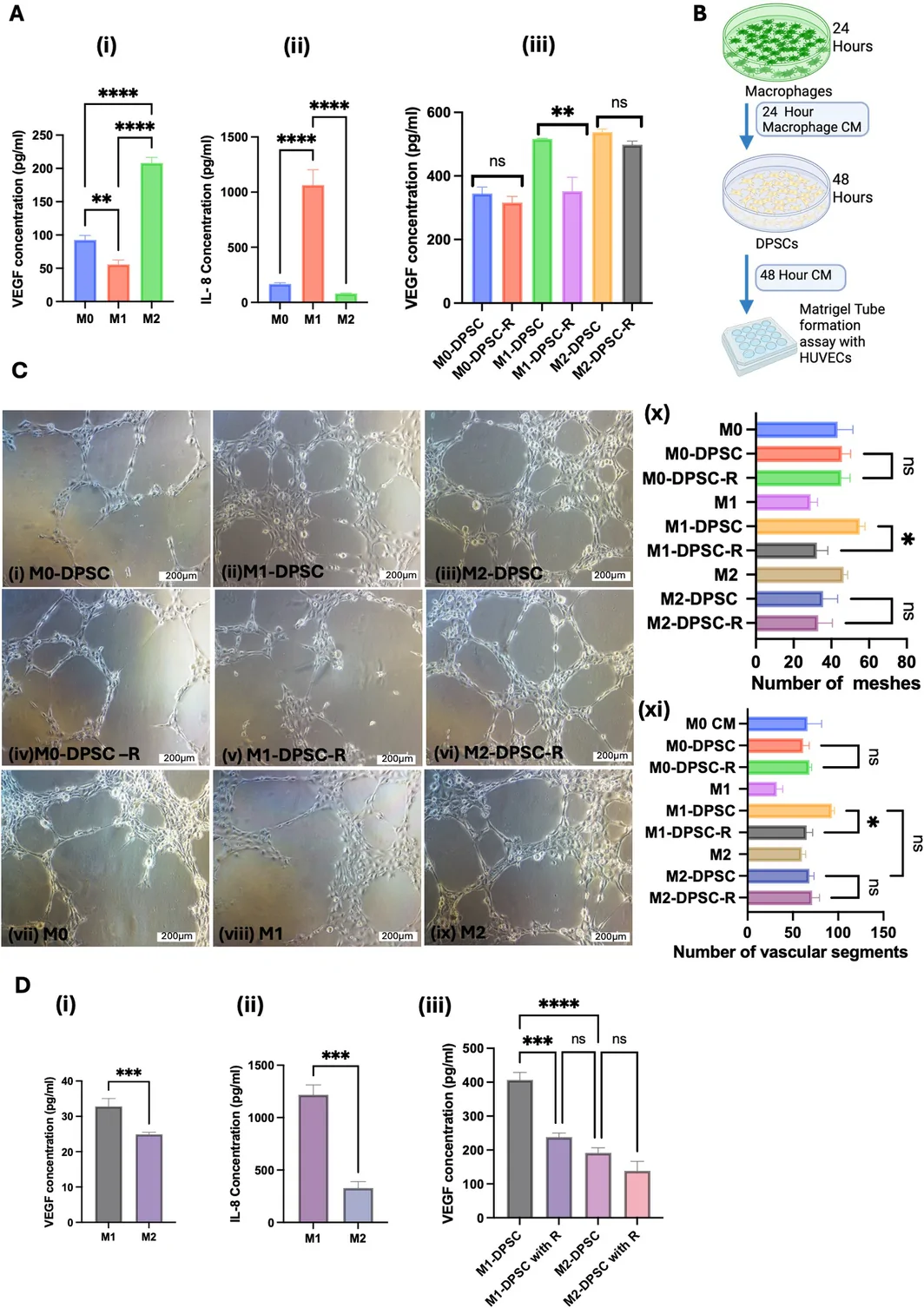
IL‐8 secreted by M1 macrophages promotes endothelial vascular tube formation by inducing DPSC VEGF secretion. (A) (i) ELISA‐VEGF and (ii) ELISA‐IL‐8 for THP‐1‐derived macrophage 24‐h CM, (iii) ELISA‐VEGF for macrophage CM (24‐h)‐treated DPSCs with or without IL‐8 inhibitor‐Reparaxin (R) for 48 h (B) Experimental workflow of CM collection. Created in BioRender. Dissanayaka, W. L. (2026) https://BioRender.com/oil0tns. (C) Matrigel angiogenesis assay with macrophage CM, macrophage CM‐treated DPSC supernatants with or without Reparaxin—8 h: (i) M0 CM‐treated DPSC CM (M0‐DPSC) (ii) M1 CM‐treated DPSC CM (M1‐DPSC) (iii) M2 CM‐treated DPSC CM (M2‐DPSC) (iv) M0 CM‐treated DPSC CM + Reparaxin (M0‐DPSC‐R) (v) M1 CM‐treated DPSC CM + Reparaxin (M1‐DPSC‐R) (vi) M2 CM‐treated DPSC CM + Reparaxin (M2‐DPSC‐R) (vii) M0 CM (M0) (viii) M1 CM (M1) (ix) M2 CM (M2). Quantification of (x) Number of meshes, and (xi) Number of vascular segments. (D) (i) ELISA‐VEGF and (ii) ELISA‐IL‐8 for PBM‐derived macrophage 48‐h CM, (iii) ELISA‐VEGF for PBM‐derived macrophage CM (48‐h)‐treated DPSCs with or without IL‐8 inhibitor‐Reparaxin (R) for 48 h *p < 0.05, **p < 0.01, ***p < 0.001, ****p < 0.0001.

Observations of increased vascular sprouting in M1‐ and M2‐macrophage‐seeded devices prompted investigation into whether angiogenic factors secreted by these macrophages contributed to this effect. Given that VEGF is a primary driver of sprouting angiogenesis, VEGF levels in macrophage‐conditioned media (CM) were quantified via ELISA. Results demonstrated that M2 macrophage CM derived from THP‐1 cells contained significantly higher VEGF concentrations (*p* < 0.0001) compared to M1 and M0 macrophage CM (Figure 5A (i)). Subsequently, based on the angiogenesis array data indicating that interleukin‐8 (IL‐8) was the most markedly upregulated cytokine in M1 macrophage CM (Figure 2E), we evaluated whether IL‐8 could modulate the angiogenic properties of DPSCs, thereby enhancing vascular sprouting. ELISA confirmation showed that IL‐8 levels were significantly elevated in M1 macrophage CM from THP‐1‐derived macrophages (*p* < 0.0001) (Figure 5A (ii)).

To elucidate the role of IL‐8 secreted by M1 macrophages in modulating VEGF secretion by DPSCs, VEGF concentrations in DPSC supernatants subjected to 24‐h macrophage‐CM treatment for 48 h, with or without the IL‐8 inhibitor Reparaxin, were quantified via ELISA. Supernatants from DPSCs treated with THP‐1‐derived M1 macrophage CM exhibited significantly elevated VEGF levels compared to those exposed to M0 CM. Furthermore, the application of Reparaxin to M1 macrophage CM resulted in a significant decrease in VEGF levels in DPSC supernatants (*p* < 0.01), a reduction not observed with M0 or M2a CM‐treated DPSCs. These findings indicate that IL‐8 within M1 macrophage CM promotes VEGF secretion by DPSCs. No significant difference in VEGF levels was detected between DPSCs treated with M1 versus M2 macrophage CM (Figure 5A (iii)). The high levels of VEGF detected in THP‐1‐derived M2‐CM‐treated DPSC supernatants could be attributed to the elevated level of VEGF that was present in M2‐CM itself, rather than those secreted by DPSCs.

To further determine whether IL‐8 in M1 macrophage CM contributes significantly in early stages of DPSC‐supported angiogenesis, DPSCs were cultured in 24‐h macrophage CM for 48 h, and a Matrigel tube formation assay was conducted using the resultant CM (Figure 5B). M1 macrophage CM‐treated DPSC CM demonstrated enhanced angiogenesis at the 8‐h time point, in terms of the number of vascular segments and number of meshes, with a significant reduction of the above parameters observed when IL‐8 inhibitor Reparaxin was added to the DPSC cultures (*p* < 0.05) (Figure 5C (i–xi)). However, the above reduction was not significant in M0 and M2 macrophage CM‐treated DPSC CM supplemented with Reparaxin groups, confirming the contribution of M1‐IL‐8‐induced DPSC VEGF in endothelial tube formation.

The ELISAs were repeated utilizing CM from PBM‐derived macrophages to treat DPSCs. Unlike THP‐1‐derived macrophages, PBM‐derived M1 macrophages exhibited significantly elevated VEGF levels compared to M2 macrophages (*p* < 0.001) (Figure 5D (i)). Consistent with the IL‐8 levels observed in THP‐1‐derived macrophages, PBM‐derived M1 macrophages also demonstrated significantly higher IL‐8 concentrations than M2 macrophage‐CM (*p* < 0.001) (Figure 5D (ii)). Comparison of the secretomes revealed that while the absolute pattern of VEGF secretion differed between the two cell types, the polarization‐dependent secretion of IL‐8 remained consistent (Figure 5A (ii) and 5D (ii)). Specifically, both THP‐1 and PBM‐derived macrophages showed a robust M1/M2 pattern for IL‐8, justifying the use of THP‐1 cells for subsequent mechanistic studies.

When DPSCs were treated with CM from PBM‐derived macrophages, those exposed to M1 macrophage‐CM showed a markedly increased VEGF secretion (*p* < 0.0001) relative to the M2 macrophage‐CM‐treated group; this elevation was significantly attenuated upon the addition of Reparaxin (*p* < 0.001) (Figure 5D (iii)). These findings corroborate the role of IL‐8 in modulating VEGF secretion by DPSCs in the context of both THP‐1‐ and PBM‐derived M1 macrophages.

### 3.5 IL‐8 Enhanced VEGF Secretion by DPSCs

While our results using Reparaxin suggest that the IL‐8/CXCR1/2 axis plays a major role in VEGF secretion by DPSCs, this receptor can be activated by several chemokines. To confirm that IL‐8 specifically drives this regulatory role, independent of other cytokines present in standard culture media, we tested the effects of recombinant IL‐8 on the secretion of VEGF by DPSCs. Significantly elevated VEGF levels were detected in all IL‐8‐treated groups compared to the control, as detected in ELISA, with the highest concentration of VEGF being observed at 0.5 ng/mL (*p* < 0.05) (Figure S2A). Significantly upregulated VEGF mRNA expression detected by qRT‐PCR in IL‐8 (0.5 ng/mL)‐treated DPSCs further confirmed the above phenomenon (*p* < 0.05) (Figure S2B). The functional effects of IL‐8‐induced VEGF secretion by DPSCs were assessed using the Matrigel assay conducted in IL‐8 (0.5 ng/mL)‐treated DPSC CM, where significantly high counts of vascular segments and junctions were observed in IL‐8‐treated DPSC CM compared to the control (*p* < 0.05) (Figure S2C,D).

Collectively, these results demonstrate that pure IL‐8 was sufficient to induce production of VEGF from DPSCs, leading to measurable functional effects. This, combined with our Reparaxin data demonstrating the necessity of the CXCR1/2 receptor, provides a complete chain of evidence for the specific role of IL‐8.

### 3.6 M1 Macrophages Enhance Angiogenic Properties in DPSCs

**FIGURE 6:**
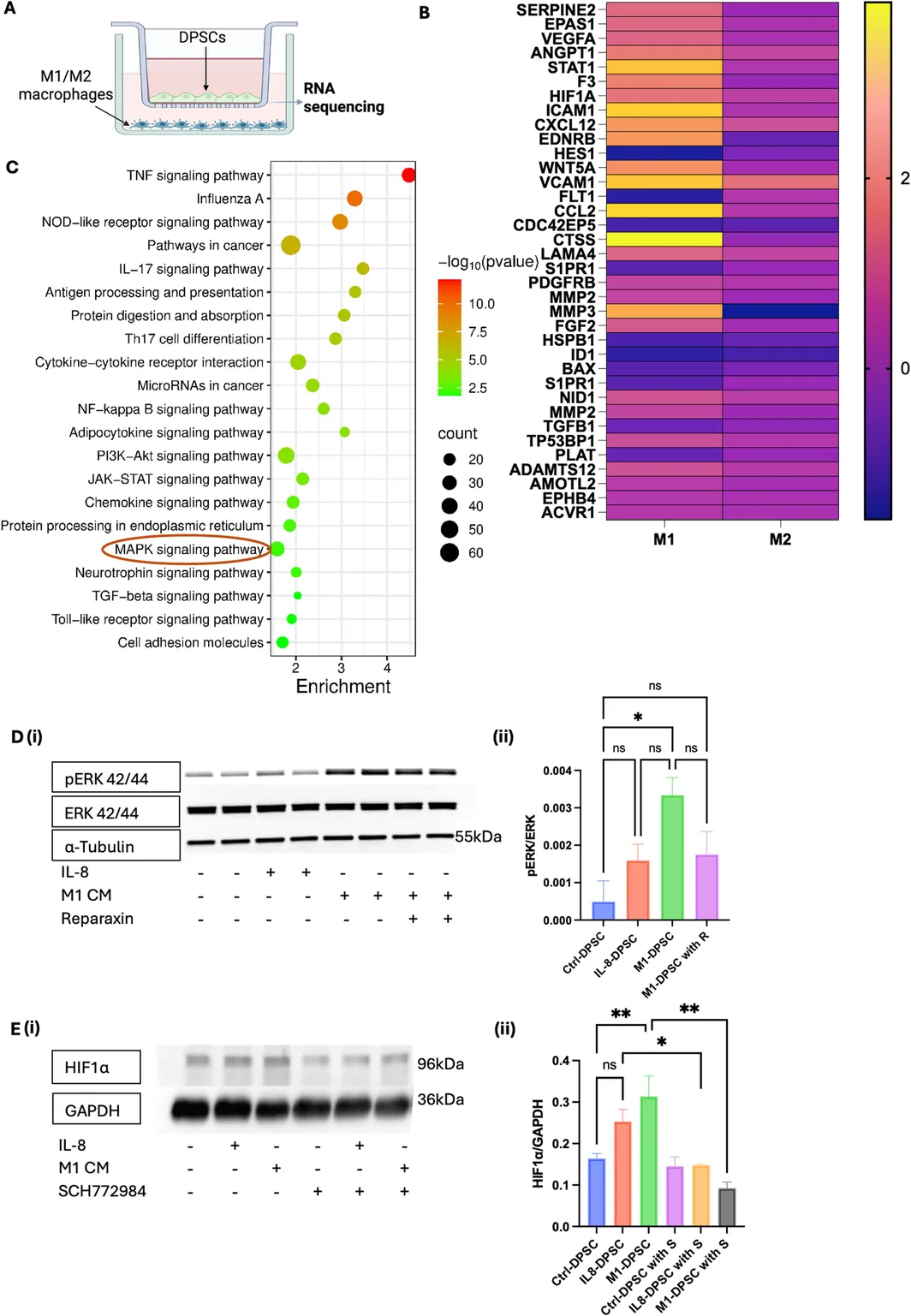
Overview of Macrophage phenotype‐induced changes in DPSC gene expression. (A) DPSC‐macrophage coculture model. Created in BioRender. Dissanayaka, W. L. (2026) https://BioRender.com/oil0tns. (B) Heatmap: Differential gene expression in DPSCs co‐cultured in M1 and M2 macrophages in angiogenesis‐related genes (compared to mono‐cultured DPSC). (C) KEGG enrichment plot of DPSCs cocultured with M1 macrophages. (D) Western blot analysis of DPSCs treated with exogenous IL‐8 and M1 CM with or without ERK inhibitor (i) and (ii) HIF1α (96 kDa) and GAPDH (36 kDa) in SCH772984. (E) Western blot analysis of DPSCs treated with IL‐8 and M1 macrophage CM with and without Reparaxin E(i) and E(ii) 42 and 44 kDa phospho‐Erk, total Erk1/2 and 55 kDa α‐Tubulin. (*n* = 3) *p < 0.05, **p < 0.01.

To assess how macrophage M1 and M2 phenotypes affect the angiogenic properties of DPSCs, a Transwell co‐culture system of PBM‐derived macrophages and DPSCs was used (Figure 6A). After 48 h of co‐culture, DPSCs were isolated, RNA‐sequenced and analysed for changes in expression compared to mono‐cultured DPSCs of a panel of 35 genes related to various steps in angiogenesis, adapted from the literature (Table S1). Among the angiogenesis‐related genes that were significantly upregulated (logFc > 1.2) post co‐culture with M1 macrophages were *Serine protease inhibitor* 2 (SERPINE2), Endothelial PAS domain protein 1 (EPAS1), VEGFA, Angiopoietin 1 (ANGPT1), Signal transducer and activator of transcription 1 (STAT1), Coagulation factor III, tissue factor (F3), HIF1α, Intercellular Adhesion Molecule 1 (ICAM1), C‐X‐C motif chemokine ligand 12 (CXCL12), Endothelin receptor type B (EDNRB), Wnt Family Member 5A (WNT5A), Vascular cell adhesion molecule 1 (VCAM1), C‐C motif chemokine ligand 2 (CCL2), Cathepsin S (CTSS) and Matrix metalloproteinase 3 (MMP3). Among them, the top 5 genes were ICAM‐1, VCAM‐1, CCL2, CTSS and STAT1 (Figure 6B).

### 3.7 IL‐8 in M1 Macrophages Enhances VEGF Secretion in DPSCs by Activating HIF‐1α via MAPK/ERK Pathway

Analysis of the protein–protein interaction network using Cytoscape identified IL6, STAT1, KRAS, HIF‐1α and ABL1 as the top five hub genes in DPSCs co‐cultured with M1 macrophages (Figure S3). Moreover, KEGG enrichment analysis identified biological signalling pathways that showed significant enrichment in the above culture (Figure 6C). MAPK/ERK, one of the most prominently upregulated pathways, and hub gene HIF1α, a key regulator of VEGF secretion in MSCs, were identified for further analysis in M1 IL‐8‐mediated VEGF secretion by DPSCs.

To examine the intracellular pathways mediating IL‐8‐induced VEGF secretion, we measured phosphorylated ERK and HIF‐1α levels in DPSCs treated with IL‐8 and M1 macrophage‐conditioned medium by Western blot analysis. The results showed that ERK phosphorylation was significantly higher in M1 macrophage CM‐treated DPSCs compared to the control group (*p* < 0.05). However, no significant difference was observed between control DPSCs and IL‐8‐treated DPSCs, which may be attributed to the waning of ERK phosphorylation by the 3‐h time point examined, as cytokine‐induced MAPK signalling is a transient process that peaks within minutes. In addition, the reduction in ERK phosphorylation following Reparaxin treatment in M1 conditioned medium‐treated DPSCs was also found to be non‐significant, which may reflect the concurrent involvement of multiple cytokines and growth factors signalling through CXCR1‐independent pathways not targeted by Reparaxin that lead to ERK phosphorylation (Figure 6D (i) and (ii)).

**FIGURE 7:**
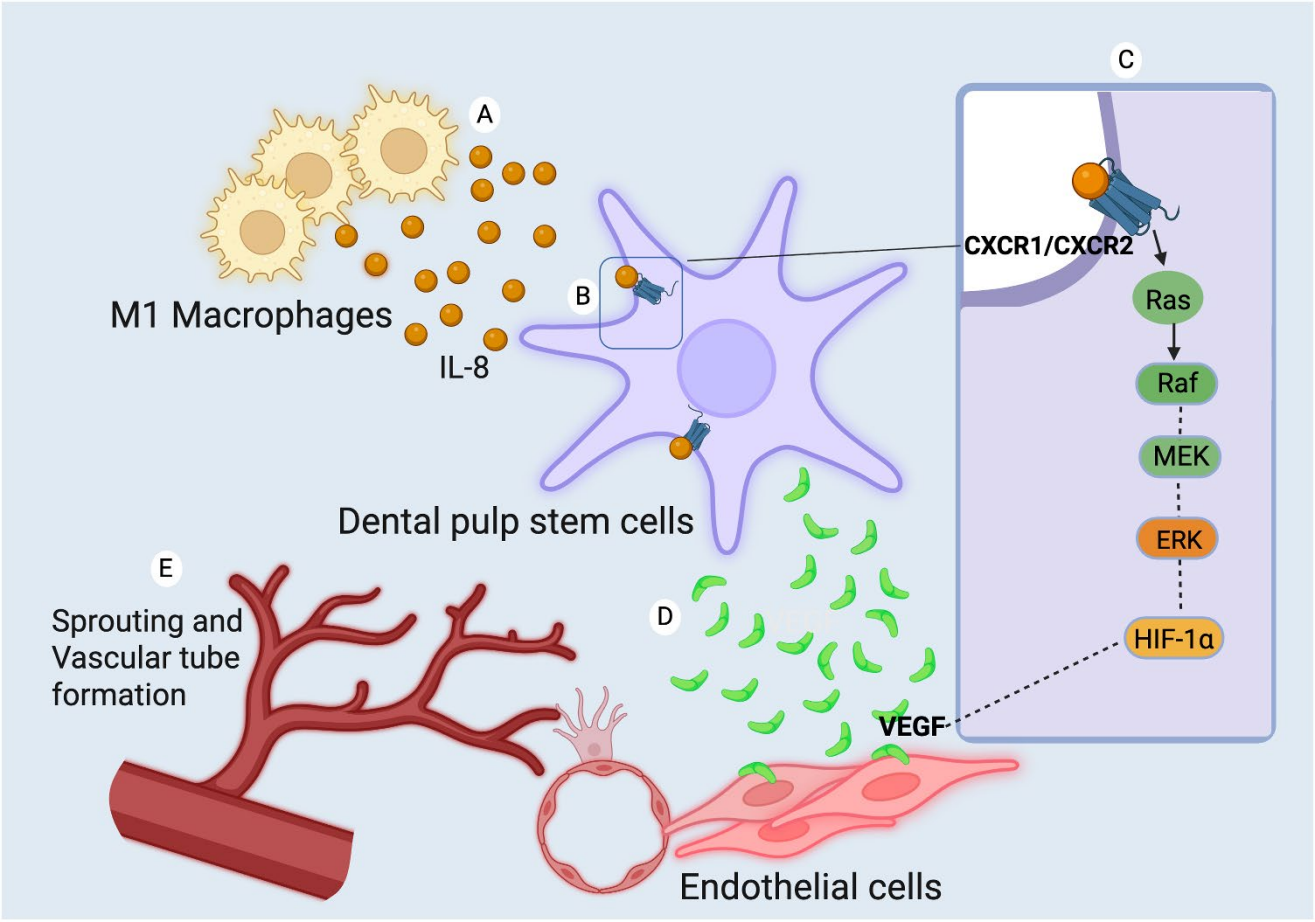
Experimental conclusion. IL‐8 secreted by M1 macrophages (A) binds to CXCR1 and CXCR2 (B) and activates the MAPK/ERK‐HIF1α pathway in DPSCs (C), inducing VEGF secretion (D), which enhances endothelial vascular tube formation and sprouting (E). Created in BioRender. Dissanayaka, W. L. (2026). https://Biorender.com/e24uenz.

HIF‐1α was detected at significantly elevated levels in M1 macrophage conditioned medium‐treated DPSCs (*p* < 0.01), with a significant reduction observed upon addition of the ERK inhibitor SCH772984 (*p* < 0.01). An increase in HIF‐1α expression in IL‐8‐treated DPSCs compared to DPSC controls was detected, although this failed to reach statistical significance, potentially reflecting the slow kinetics of IL‐8‐driven HIF‐1α accumulation that typically peaks 18 h post‐stimulation. However, a significant reduction in HIF‐1α expression was observed in IL‐8‐treated DPSCs following treatment with the ERK inhibitor SCH772984 (*p* < 0.05), suggesting that the ERK/HIF‐1α axis is operative in IL‐8/CXCR1/2 signalling (Figure 6E (i) and (ii)). As the upregulation of HIF‐1α is strongly linked with enhanced VEGF expression, the above evidence suggests MAPK/ERK‐HIF‐1α as a candidate pathway involved in IL‐8‐induced upregulation of VEGF in DPSCs. Figure 7 outlines the key points of the experimental conclusion.

## 4 Discussion

The phenotypic variants of macrophages, which exist primarily as M1 and M2, play distinct roles across various stages of inflammation, mediated by crosstalk among multiple cell types and by the secretion of a wide array of growth factors and cytokines. M1 macrophages secrete pro‐inflammatory cytokines such as IL‐8, TNF‐α, IL‐12 and IL‐1β that aid in combating pathogens by promoting phagocytosis, recruiting additional immune cells and neutralizing bacterial products (Akhtari et al. 2021; Deng et al. 2025; Jaguin et al. 2013; Liao et al. 2019). At later reparative stages of inflammation, pro‐regenerative M2 predominate and M1 macrophages polarize into M2 macrophages (Gu and Kaneko 2019), promoting resolution of inflammation and repair via secretion of anti‐inflammatory and pro‐regenerative cytokines and growth factors such as IL‐10, PDGF and TGFβ1 (Heinrich et al. 2017; Shapouri‐Moghaddam et al. 2018). This study provides novel evidence that the interaction between pro‐inflammatory M1 macrophages and DPSCs orchestrates a pronounced pro‐angiogenic response.

Recent studies claim that M1 macrophages possess certain pro‐regenerative properties. One study reported that M1 macrophage CM enhanced the proliferation and adipogenic differentiation of bone marrow mesenchymal stem cells (BMMSCs), with an increased production of ECM proteins in cell sheets (He et al. 2018). Previous research also indicates that M1 macrophages play a critical role in post‐injury muscle repair, with hindered accumulation leading to impaired healing (Miyazaki et al. 2023). Further, in coculture systems, M1 macrophages have been shown to exert a pro‐osteogenic effect, enhancing bone mineralization by MSCs (Lu et al. 2017).

In the current study, we used macrophages derived from two sources: human THP‐1 cells and human PBMs, with THP‐1 cells as the primary source. The advantages of using THP‐1 cells include the convenience of culture, consistency that allows for maintaining reproducibility across studies and simplified experimental setups as opposed to PBM‐derived macrophages, where donor variability may result in inconsistent results (Hoppenbrouwers et al. 2022). On the contrary, THP‐1 cells may not fully resemble PBM‐derived macrophages functionally and phenotypically (Graney et al. 2020). Especially where genetic analyses are involved, PBM can be considered a more appropriate source, considering that THP‐1 cells are a malignant cell line.

In the current study, VEGF levels were detected in higher amounts in M1 macrophages compared to M2 macrophages in PBM‐derived macrophages, as was reported in previous studies (Graney et al. 2020), whereas in THP‐1‐derived macrophages, VEGF was detected in higher amounts in M2 macrophages compared to M1 macrophages. The observed differences in VEGF secretion magnitude between THP‐1 and primary macrophages are a known characteristic of these models (Lund et al. 2016; Tedesco et al. 2018). PMA‐differentiated THP‐1 cells develop a high pro‐inflammatory baseline through intense Protein Kinase C (PKC) and NF‐κB activation (Kim et al. 2022; Song et al. 2015), potentially causing desensitization to subsequent M1 stimuli and a relatively lower M1‐VEGF response. In contrast, M‐CSF‐differentiated PBMs undergo more physiological maturation, maintaining robust NF‐κB/HIF‐1α‐driven transcriptional responses to M1 polarization and higher VEGF levels, while M2 polarization activates the STAT6/PPAR‐γ axis, yielding comparatively lower VEGF secretion (Kim et al. 2022; Mantsounga et al. 2022; Rius et al. 2008; Song et al. 2015; Tannahill et al. 2013). Importantly, while the patterns of VEGF secretion varied, the secretion pattern of IL‐8, the primary cytokine of interest in this study, was highly consistent across both cell types. Furthermore, both models exhibited identical patterns of standard M1/M2 phenotypic markers, confirming successful and consistent polarization in both systems.

In the present study, we utilized a microfluidic system for recapitulation of the in vivo scenario involved in the crosstalk between DPSCs, HUVECs and macrophages within a fibrin hydrogel matrix. Microfluidic devices provide the benefit of step‐by‐step observation of the process in a controlled environment that closely mimics the physiological conditions (Gupta et al. 2016).

The markedly enhanced pro‐angiogenic features observed in M1 macrophage‐seeded models in the current study could be explained by the significantly high concentration of VEGF secreted by DPSCs (as detected by ELISA) under the influence of IL‐8 in the M1 macrophage secretome. Additionally, they may be influenced by the direct angiogenic effects of IL‐8 on HUVECs as well as the effects of many other growth factors and cytokines secreted by M1 macrophages, including TNF‐α, which is reported to increase VEGF secretion by MSCs (Zubkova et al. 2016). The results of the above microfluidic assay are consistent with the findings of a 3D triculture assay conducted on PLGA scaffolds, where M1‐seeded MSC supported vascular constructs displayed increased vascularization relative to both the M0 control and constructs without macrophages, but not relative to M2‐seeded constructs (Graney et al. 2020). For distinctly assessing the angiogenic effects of IL‐8 in macrophage CM, the Matrigel assay was chosen over the microfluidic assay in the current study, as the former allows specific assessment of the cell migration and tube formation stages of angiogenesis promoted by the growth factors of the CM over a span of 6–24 h (Kanugula et al. 2024).

When the density of seeded macrophages in the microfluidic systems was increased, a significant reduction in vascularization was observed. This could be attributed to the higher degree of cytotoxicity that may have prevailed in the micro‐environment due to the expression of reactive oxygen species, inducible nitric oxide synthase (iNOS) and pro‐inflammatory cytokines such as IL‐12 by M1 macrophages (Zhang et al. 2024). This was revealed through the significantly high number of dead cells detected with propidium iodide. Bindal et al. in their study discovered that in response to LPS stimulation, DPSCs secrete increased levels of pro‐inflammatory mediators such as IL‐6 and IL‐8 (Bindal et al. 2018). However, Xu et al. reported that the effect of LPS on DPSCs is concentration and duration‐dependent, with lower LPS concentrations promoting dentinogenic differentiation (Xu et al. 2022). Similar findings have been reported in relation to the pro‐inflammatory cytokine TNF‐α, where odontogenic differentiation of DPSCs was promoted at lower concentrations (Kang et al. 2022). Reports also indicate that TNF‐α promotes migration and osteogenic differentiation of DPSCs (Feng et al. 2013) and, within short‐term exposure, affects the cell viability of DPSCs, while enhancing the expression of VEGF with long‐term exposure, giving rise to increased proliferation (Boyle et al. 2014). Further, TNFα was also found to promote angiogenesis in endothelial cell‐DPSC co‐cultures (An et al. 2019). Taken together, the dual nature of the DPSC response highlights how the inflammatory microenvironment profoundly dictates the tissue's regenerative capacity. While acute, high‐level inflammation causes tissue destruction, low‐level or transient inflammatory stimuli can actually prime DPSCs, enhancing their proliferation, migration and angiogenic potential. This biphasic behaviour demonstrates that the ultimate functional outcome of DPSCs is highly sensitive to the severity and duration of inflammation within the pulpal niche. Consequently, successful vital pulp therapy relies on modulating, rather than entirely suppressing, these inflammatory signals to successfully transition the tissue from a defensive phase to a reparative state.

In this study, we have demonstrated that DPSCs secrete higher levels of VEGF in an inflammatory milieu, where it plays a multifaceted role. VEGF is found to induce the proliferation of not only endothelial cells but also other dental pulp cells. In addition, VEGF can promote differentiation of DPSCs and transition of M1 macrophages to an anti‐inflammatory M2 phenotype (Mullins et al. 2019; Wheeler et al. 2018). Further, VEGF can enhance vascular permeability (Chen et al. 2012), facilitating extravasation and diapedesis of inflammatory cells, such as lymphocytes, neutrophils and monocytes (Botero et al. 2003; Piwowarczyk et al. 2025; Wheeler et al. 2018). However, the effects of increased vascular permeability could lead to an increase in intra‐pulpal pressure, which, if not maintained at safe levels, could result in ischaemia and tissue necrosis (Bryniarska‐Kubiak et al. 2024; Botero et al. 2003; Matsushita et al. 1999). Overall, it can be speculated that the inflammatory regenerative effects may only be conditionally favourable for pulp repair.

When DPSCs were co‐cultured with M1 macrophages, the angiogenesis‐related genes ICAM‐1, VCAM‐1, CCL2, CTSS and STAT1 were identified as the top five upregulated genes in DPSCs and are implicated in key angiogenic and inflammatory mechanisms. ICAM‐1 is known to play a major role in the context of polymorphonuclear leukocyte‐induced angiogenesis, where it acts as a signalling receptor that upregulates ETS proto‐oncogene 1 (Ets‐1) expression, which is capable of inducing the expression of matrix metalloproteinases and integrin beta3 in endothelial cells (Yasuda et al. 2002). VCAM‐1 is also secreted in response to inflammatory cytokines and is known to interact with integrins to promote further recruitment of monocytes and macrophages that can secrete pro‐angiogenic growth factors such as VEGF and Fibroblast growth factor (FGF) (Garmy‐Susini and Varner 2008). Further, CCL2 is known as a molecule that is primarily involved in the recruitment of immune cells to the sites of inflammation. Its effects on endothelial cells include promoting their proliferation, tube formation and migration (Lu and Kang 2009; Stamatovic et al. 2006). CTSS is a lysosomal cysteine protease that contributes to extracellular matrix remodelling and immune regulation. Its role in angiogenesis includes activation of pro‐angiogenic factors such as VEGF and degradation of extracellular matrix components, for example, Collagen and Elastin, thereby facilitating migration and invasion of endothelial cells (Gocheva et al. 2010; Wang et al. 2006). While all 4 of the above are primarily involved in inflammatory angiogenesis, STAT1 presents a controversial and context‐dependent role in angiogenesis. Even though its role is identified in inhibition of angiogenesis via down‐regulation of pro‐angiogenic factors such as VEGF, certain studies claim that STAT1 can promote angiogenesis by enhancing endothelial cell survival and proliferation, particularly in response to growth factors like Epidermal growth factor (EGF) (Kujawski et al. 2008).

In our study, we demonstrated that M1 macrophage‐DPSC interaction enhances angiogenesis via IL‐8. Although IL‐8 is classically recognized as a pro‐inflammatory cytokine responsible for activating and recruiting neutrophils to injury sites (de Oliveira et al. 2013), emerging evidence highlights its conditional pro‐regenerative capacity. Specifically, IL‐8 has been shown to induce angiogenesis (Li et al. 2005; Li et al. 2003), as well as promote the osteogenic and chondrogenic differentiation of BMMSCs (Yang et al. 2018; Wang et al. 2023).

Crucially, the functional outcome, whether pro‐inflammatory or pro‐regenerative, appears to be dictated by the local concentration of the cytokine. For instance, while high clinical levels of IL‐8 drive acute tissue destruction, low concentrations (e.g., 10 ng/mL) can polarize neutrophils toward a pro‐healing N2 phenotype that downregulates pro‐inflammatory signals (such as TNF and IFN‐γ) and secretes SDF‐1α to recruit regenerative progenitor cells (Cai et al. 2021). To target this regenerative window, we evaluated a range of exogenous IL‐8 concentrations in DPSC cultures, identifying 0.5 ng/mL as the optimal dose for maximizing VEGF secretion. This finding aligns with previous BMMSC models reporting IL‐8‐mediated VEGF induction (Hou et al. 2014), though our system achieves this effect at a significantly lower threshold as opposed to the 25‐200 ng/mL range used by Hou et al. It has been reported that in the dental pulp, the IL‐8 levels in reversible pulpitis range from 187.5 to 310.0 pg/mL whereas in irreversible pulpitis this range from 1036.7 to 2177.6 pg/mL which further justifies the low concentration of IL‐8 used in our study and the fact that lower IL‐8 may be detected in reversible pulpitis where M1 macrophages are found in scarce amounts indicative of the potential for pulp regeneration (Nawal et al. 2024).

A common challenge in cytokine research is the potential for synergistic interactions between multiple factors in the cellular microenvironment (Akdis et al. 2016). Our findings also suggest that while M1 macrophages secrete a pleiotropic mix of angiogenic factors, the specific regulatory role we observed is driven by the IL‐8/CXCR1/2 axis. Other factors detected in our array, such as CXCL16 (which signals through CXCR6) (Gruber et al. 2017) and HB‐EGF (which signals through EGFR) (Singh et al. 2016), do not interact with the receptors targeted by Reparaxin. While CXCR1 and CXCR2 serve as receptors for various ligands (Stillie et al. 2009), IL‐8 is widely recognized as the primary and most potent pro‐angiogenic ligand for this axis in humans (Brat et al. 2005; Dwyer et al. 2012; Garcia et al. 2025; Li et al. 2005). Additionally, in similar M1 macrophage secretomes, IL‐8 is typically the dominant driver of CXCR1/2‐mediated effects (Engström et al. 2014; Kaur and Singh 2013; Tarique et al. 2015). Moreover, several co‐secreted cytokines amplify this effect by upregulating IL‐8 production itself, reinforcing IL‐8 as the central node (Rathanaswami et al. 1993; Sarir et al. 2010; Thomas et al. 2000). Furthermore, our recombinant exogenous IL‐8 data confirm that IL‐8 alone, at concentrations mirroring those in our M1 media, is sufficient to reproduce the effect in a defined system.

As identified in the current study, the M1 CM‐derived IL‐8‐mediated VEGF induction involves the MAPK/ERK/HIF‐1α signalling cascade downstream of CXCR1/2 engagement, consistent with the well‐identified link between HIF‐1α activation and VEGF transcription (Forsythe et al. 1996). For intracellular signalling pathway analysis, the 3‐h timepoint was selected as it corresponds to the established peak of ERK‐driven HIF‐1α stabilization (Jung et al. 2002), a selection further supported by the significant HIF‐1α elevation observed in M1 CM‐treated DPSCs at this timepoint. Although exogenous recombinant IL‐8 treatment significantly increased downstream VEGF secretion by DPSCs at 48 h, the absence of significant intracellular signalling effector upregulation at the 3‐h timepoint requires mechanistic interpretation.

In mesenchymal cells, ERK activation typically peaks within 5–15 min of stimulation and returns to baseline after 120 min (Gharibi et al. 2012; Wortzel and Seger 2011). Therefore, the lack of significant ERK activation by exogenous IL‐8, at 3 h likely reflects the waning of the phosphorylation signal rather than its absence. Further, the non‐significant upregulation of HIF‐1α in IL‐8‐treated DPSCs at the 3‐h time point reflects a quantitative limitation imposed by the kinetics of IL‐8‐induced normoxic HIF‐1α stabilization. Unlike potent inflammatory stimuli or multi‐cytokine environments, IL‐8; a single cytokine acting under normoxic conditions induces HIF‐1α stabilization slowly, with peak accumulation reported at approximately 18 h rather than 3 h (Abdi Sarabi et al. 2022). However, the significant reduction of HIF‐1α by the ERK inhibitor SCH772984 in IL‐8‐treated DPSCs, despite the non‐significant HIF‐1α upregulation in IL‐8 treated DPSCs suggests that the ERK/HIF‐1α axis is operative downstream of IL‐8/CXCR1/2 signalling at this timepoint, and that peak HIF‐1α accumulation is yet to be reached.

As shown in our angiogenesis array, M1‐conditioned media contain multiple potent ERK‐activating factors such as VEGF, PDGF and TNFα. Because these factors activate ERK through their own cognate receptors (VEGFR, PDGFR and TNFαR) (Gharibi et al. 2012; Ma et al. 2019; Narasimhan et al. 2009), the inhibition of CXCR1/2 by Reparaxin, which only addresses the IL‐8‐mediated component of total ERK phosphorylation, is likely masked by the redundant signalling of other cytokines. Moreover, IL‐8/CXCR1/2 engagement is known to concurrently activate multiple downstream signalling pathways, including PI3K/AKT and NF‐κB, in addition to MAPK/ERK (Hou et al. 2014; Martin et al. 2009). While the individual contribution of each pathway may not independently reach statistical significance, their combined activation could collectively account for the magnitude of VEGF secretion observed.

Taken together, the significant reduction of VEGF secretion by Reparaxin, the ability of exogenous IL‐8 alone to significantly induce VEGF, the significant ERK and HIF‐1α elevation in M1 CM‐treated DPSCs, and the significant ERK inhibitor‐mediated HIF‐1α reduction in both M1 CM‐ and IL‐8‐treated DPSCs collectively suggest the MAPK/ERK/HIF‐1α axis as a contributory signalling pathway mediating IL‐8‐induced VEGF secretion in DPSCs.

As our study has focused only on the paracrine interaction between macrophages and DPSCs, further studies are required to identify the effects of direct interactions among dental pulp cells, macrophages and other cytokines and growth factors that predominate in the spectrum of events associated with pulpitis, and to assess their cumulative effects in the above microenvironment. More importantly, an in vivo experimental setup may help create a clearer, more comprehensive picture of the involvement of other immune cells in pulpitis. Furthermore, given that Reparaxin blocks the shared CXCR1/2 receptors, the potential synergistic contributions of other ELR^+^ CXC chemokines to DPSC‐derived VEGF secretion cannot be entirely excluded by the current study. In addition, given the transient nature of intracellular effectors involved in the pathways assessed, the use of a single time point, as in this study, may not fully capture the mechanistic sequence. Assessment of multiple timepoints in future studies would therefore enable a more comprehensive temporal characterization of the IL‐8/CXCR1/2/MAPK/ERK/HIF‐1α signalling cascade in DPSCs.

Another limitation of this study is the relatively small cohort of 4 healthy blood donors used in the PBMC‐derived macrophage experiments. While this sample size is consistent with established primary cell co‐culture models (Graney et al. 2020; Keramati et al. 2025), it may not fully capture the broader spectrum of human biological diversity. To mitigate the impact of donor heterogeneity and ensure reproducibility, we employed strict inclusion criteria regarding age and health status and utilized a monocyte pooling strategy. Nonetheless, future studies involving larger, more diverse populations, including variations in age, ethnicity and underlying health conditions, would be beneficial to confirm the generalisability of our findings across the wider clinical population.

Looking forward, several future research prospects emerge from this study to translate these cellular interactions into clinical vital pulp therapies. While our in vitro model establishes the critical role of the M1 macrophage‐DPSC axis via CXCR1/2 signalling, in vivo animal models of pulpitis are required to validate these temporal dynamics within a complex biological root canal system. Future investigations should focus on the strategic biomaterial‐based delivery of ultra‐low dose IL‐8 or CXCR1/2 modulators. Developing injectable, biocompatible hydrogels capable of sequentially releasing these signalling molecules could allow clinicians to precisely control macrophage polarization and DPSC recruitment at carious exposure sites.

## 5 Conclusion

In conclusion, this study showed that M1 macrophages promote angiogenesis via IL‐8‐induced DPSC VEGF secretion, in a density‐dependent manner, through CXCR1/2‐MAPK/ERK‐HIF‐1α signalling. These findings have direct clinical implications for regenerative endodontics, suggesting that the success of vital pulp therapy depends on a finely tuned inflammatory microenvironment. Our results indicate that M1 macrophages are not merely markers of disease but are active participants in early repair. This shifts the therapeutic focus from total anti‐inflammatory intervention toward the strategic modulation of the M1 activity to promote vascular‐driven healing in inflamed pulp tissue.

## Author Contributions

Dineshi Sewvandi Thalakiriyawa: Data acquisition, data analysis, visualization, writing – original draft. Mohamad Koohimoghadam: Data analysis. Hu Mingxin and Wang Hong: Data acquisition. Colman McGrath: Supervision, writing – review and editing. Waruna Lakmal Dissanayaka: Conceptualization, supervision, project administration, funding acquisition, writing – review and editing. All authors have read and agreed to the published version of the manuscript.

## Funding

This work was supported by the General Research Fund (Project Number GRF17119524), Research Grants Council, University Grants Committee of Hong Kong, and the Seed Fund for PI Research—Basic Research (Project code 2302101543), The University of Hong Kong, Hong Kong.

## Ethics Statement

All biological procedures described were performed in accordance with the regulations and guidelines of the University of Hong Kong. Approval from the Institutional Review Board of the University of Hong Kong was obtained for procedures involving human participants.

## Conflicts of Interest

The authors declare no conflicts of interest.

## Supporting information


**Figure S1:** (A) Characterization of macrophage phenotypes. ELISA‐TNF‐α in (i) THP‐1 cell‐derived macrophage CM (ii) PBM‐derived macrophage CM. ***p < 0.001, ****p < 0.0001.
**Figure S2:** (A) ELISA‐VEGF in IL‐8‐treated DPSC CM. (B) mRNA expression of VEGF in IL‐8‐treated DPSCs. (C) Matrigel assay in (i) IL‐8 treated DPSC CM 48 h (ii) DPSC CM (Control) 48 h (D) (i) Number of vascular segments at 8 h (ii) Number of Junctions at 8 h. *p < 0.05***p < 0.001, ****p < 0.0001.
**Figure S3:** Human protein interaction network constructed in Cytoscape software V2.2. The top 5 hub genes identified using the plugin Centiscape, HIF‐1α, KRAS, ABL1, IL‐6 and STAT1, are indicated in red.
**Table S1:** List of genes involved in angiogenesis assessed in DPSCs cocultured with PBM‐derived macrophages and References for Table S1.

**Appendix S1:** PRILE 2021 Checklist of items to be included when reporting laboratory studies in Endodontology.

## Data Availability

The data that support the findings of this study are available from the corresponding author upon reasonable request.
