# Supplementary material for: The Interplay of M1 Macrophages and Dental Pulp Stem Cells Promotes Angiogenesis Through IL‐8‐Dependent VEGF Regulation: An In Vitro Study
Source: Int Endod J. 2026 Jun 24;59(10):2143–63. doi: 10.1111/iej.70198 (PMC13569155; doi:10.1111/iej.70198)
Supplement: Supplementary file 2 [file IEJ-59-2143-s002.docx]

**Supplementary results**

**Figure S.1:** (A) Characterization of macrophage phenotypes. ELISA-TNF-α in (i)THP-1 cell-derived macrophage CM (ii) PBM-derived macrophage CM. ***p<0.001,****p<0.0001

**Figure S.2.** (A) ELISA-VEGF in IL-8 treated DPSC CM (B) mRNA expression of VEGF in IL-8 treated DPSCs (C) Matrigel assay in (i) IL-8 treated DPSC CM 48hrs (ii) DPSC CM (Control) 48hrs (D) (i) Number of vascular segments at 8 hours (ii) Number of Junctions at 8 hours. * p<0.05***p<0.001,****p<0.0001

Figure S.3 Human protein interaction network constructed in Cytoscape software V2.2. The top 5 hub genes identified using the plugin Centiscape, HIF-1α, KRAS, ABL1, IL-6, and STAT1, are indicated in red.

Table S.1: List of genes involved in angiogenesis assessed in DPSCs cocultured with PBM-derived macrophages

|  | Gene | Function in Angiogenesis |
| --- | --- | --- |
| 1 | SERPIN2 | - Implicated in both promoting and inhibiting angiogenesis depending on the context.   Inhibits uPA to influence ECM remodeling and endothelial signaling (Yang et al., 2018) |
| 2 | EPAS1 | - Promotes angiogenesis during hypoxia by activating genes for VEGF, VEGFR1, VEGFR2, and Tie2 to drive vessel growth and maturation. (Takeda et al., 2004) |
| 3 | VEGFA | - Stimulates endothelial cell proliferation, migration, and survival to form new blood vessels in both normal and disease states (Hicklin and Ellis, 2005). |
| 4 | ANGPT1 | - Activates the Tie2 receptor to promote vascular stability, maturation, and endothelial cell survival (Gavard et al., 2008; Maisonpierre et al., 1997). |
| 5 | STAT1 | - Plays a controversial and context dependent role in angiogenesis. Even though its role is identified in inhibition of angiogenesis via down-regulation of pro-angiogenic factors such as VEGF, certain studies claim that STAT1 can promote angiogenesis by enhancing endothelial cell survival and proliferation, particularly in response to growth factors like Epidermal growth factor (EGF)(Kujawski et al., 2008). |
| 6 | F3 | - Promotes angiogenesis by upregulating VEGF and IL-8, and its spliced forms stimulate signaling via endothelial integrins (Huang et al., 2017). |
| 7 | HIF1A | - Upregulates angiogenic factors like VEGF, bFGF, and angiopoietins to promote vessel formation, especially under hypoxic conditions (Cao et al., 2009; Wen et al., 2019; Wu et al., 2014). |
| 8 | ICAM1 | - Facilitates polymorphonuclear leukocyte-induced angiogenesis by upregulating Ets-1, which enhances MMP and integrin expression in endothelial cell (Yasuda et al., 2002). |
| 9 | CXCL12 | - Promotes angiogenesis by binding CXCR4 to mobilize endothelial cells and recruit endothelial progenitor cells (Guo et al., 2017; Teicher and Fricker, 2010). |
| 10 | EDNRB | - Mediates endothelin-1 effects to stimulate endothelial cell proliferation and migration, promoting tumor angiogenesis (Salani et al.,2000). |
| 11 | HES1 | - A Notch signaling effector, regulates endothelial cell behavior to influence vessel formation and stabilization (Yao et al., 2017). |
| 12 | WNT5A | - Promotes angiogenesis by stimulating pro-angiogenic factor release, increasing VEGF expression, and regulating collective endothelial cell migration (Kikuchi et al., 2014; Zhao et al., 2021; Tian et al., 2022; Carvalho et al., 2019; Padwal et al., 2020) |
| 13 | VCAM1 | - Enhances migration and angiogenic capacity of bone marrow mesenchymal stem cells. (Shao et al., 2023) |
| 14 | FLT1(VEGFR) | - Plays a dual role in angiogenesis as it binds to VEGF and placental growth factor (PlGF). It functions both as a decoy receptor that limits excessive angiogenic signalling from VEGF and as a signalling receptor that is involved in the recruitment of hematopoietic progenitor cells, thus influencing the balance of angiogenesis in both physiological and pathological conditions (Kaplan et al., 2005; Olsson et al., 2006) |
| 15 | CCL2 | - Promotes angiogenesis by recruiting immune cells and directly stimulating endothelial cell proliferation, migration, and tube formation, tube formation and migration. (Lu and Kang, 2009; Stamatovic et al., 2006) |
| 16 | CDC42EP5 | - Regulates actin dynamics and endothelial cell motility to facilitate new blood vessel formation(Farrugia et al., 2020) |
| 17 | CTSS | - Promotes angiogenesis by degrading ECM components and activating pro-angiogenic factors like VEGFof endothelial cells.(Gocheva et al., 2010; Wang et al., 2006) |
| 18 | LAMA4 | - Facilitates angiogenesis by promoting endothelial cell attachment, migration, and tube formation..(Moore et al., 2015; Shan et al., 2015) |
| 19 | S1PR1 | - Promotes vascular stability and endothelial barrier function while also restricting excessive sprouting(Wang et al., 2022a) |
| 20 | PDGFRB | - Mediates pericyte proliferation and migration, which is essential for vessel stabilization.(Raica and Cimpean, 2010) |
| 21 | MMP-2 | - Promotes angiogenesis by degrading the ECM, releasing growth factors, and influencing endothelial cell signaling.(Stetler-Stevenson, 1999) |
| 22 | MMP-3 | - Acts as a pro-angiogenic factor by remodeling tissue and facilitating endothelial cell migration(Deryugina and Quigley, 2010). |
| 23 | FGF2 | - Stimulates endothelial cell proliferation, migration, and survival, and recruits pericytes to stabilize new vessels. (Hattori et al., 2024) |
| 24 | HSPB1 | - Has a dual role, promoting angiogenesis via cytoskeleton remodeling but yielding inhibitory fragments when cleaved by MMP9 (Lee et al., 2012). |
| 25 | ID1 | - Promotes angiogenesis by blocking endothelial cell differentiation and inhibiting anti-angiogenic factors (Volpert et al., 2002). |
| 26 | BAX | - Inhibits angiogenesis by promoting apoptosis in endothelial cells) of endothelial cells (Chura-Chambi et al., 2014; Sheibani et al., 2024). |
| 27 | SRPR1 | - Promotes angiogenesis by enhancing endothelial cell migration, proliferation, and barrier function (Wang et al., 2022). |
| 28 | NID1 | - Can promote angiogenesis by enhancing endothelial adhesion and survival but also acts as an anti-angiogenic factor in mature vessels (Cheng et al., 2022). |
| 29 | TGFB1 | - Has dual roles, promoting vessel sprouting in some contexts and inhibiting it to aid maturation in others (Goumans and ten Dijke, 2018). |
| 30 | TP53BP1 | - Generally acts as an angiogenesis inhibitor, particularly in cancer (Li et al., 2013). |
| 31 | PLAT | - Promotes angiogenesis by degrading the ECM to facilitate endothelial cell migration (Noh et al., 2021). |
| 32 | ADAMTS12 | - Can inhibit angiogenesis as a tumor suppressor but may promote it in specific cancers by upregulating VEGF (Jiang et al., 2023). |
| 33 | AMOTL2 | - Crucial for angiogenesis by regulating endothelial cell migration, polarity, and proliferation (Wang et al., 2011). |
| 34 | EPHB4 | - Promotes angiogenesis and vessel patterning through interactions with EphrinB2 (Erber et al., 2006). |
| 35 | ACVR1 | - Promotes angiogenesis by suppressing p21 to enhance endothelial cell proliferation.(Pak et al., 2024). |

**References for Table S.1.**

Cao, D., Hou, M., Guan, Y.-S., Jiang, M., Yang, Y., Gou, H.-F., 2009. Expression of HIF-1alpha and VEGF in colorectal cancer: association with      clinical outcomes and prognostic implications. BMC Cancer 9, 432. https://doi.org/10.1186/1471-2407-9-432

Carvalho, J.R., Fortunato, I.C., Fonseca, C.G., Pezzarossa, A., Barbacena, P., Dominguez-Cejudo, M.A., Vasconcelos, F.F., Santos, N.C., Carvalho, F.A., Franco, C.A., 2019. Non-canonical Wnt signaling regulates junctional mechanocoupling during      angiogenic collective cell migration. Elife 8. https://doi.org/10.7554/eLife.45853

Cheng, P., Cao, T., Zhao, X., Lu, W., Miao, S., Ning, F., Wang, D., Gao, Y., Wang, L., Pei, G., Yang, L., 2022. Nidogen1-enriched extracellular vesicles accelerate angiogenesis and bone      regeneration by targeting Myosin-10 to regulate endothelial cell adhesion. Bioact. Mater. 12, 185–197. https://doi.org/10.1016/j.bioactmat.2021.10.021

Chura-Chambi, R.M., Bellini, M.H., Jacysyn, J.F., Andrade, L.N., Medina, L.P., Prieto-da-Silva, A.R.B., Amarante-Mendes, G.P., Morganti, L., 2014. Improving the therapeutic potential of endostatin by fusing it with the      BAX BH3 death domain. Cell Death Dis. 5, e1371. https://doi.org/10.1038/cddis.2014.309

Deryugina, E.I., Quigley, J.P., 2010. Pleiotropic roles of matrix metalloproteinases in tumor angiogenesis:      contrasting, overlapping and compensatory functions. Biochim. Biophys. Acta 1803, 103–120. https://doi.org/10.1016/j.bbamcr.2009.09.017

Erber, R., Eichelsbacher, U., Powajbo, V., Korn, T., Djonov, V., Lin, J., Hammes, H.-P., Grobholz, R., Ullrich, A., Vajkoczy, P., 2006. EphB4 controls blood vascular morphogenesis during postnatal angiogenesis. EMBO J. 25, 628–641. https://doi.org/10.1038/sj.emboj.7600949

Farrugia, A.J., Rodríguez, J., Orgaz, J.L., Lucas, M., Sanz-Moreno, V., Calvo, F., 2020. CDC42EP5/BORG3 modulates SEPT9 to promote actomyosin function, migration,      and invasion. J. Cell Biol. 219. https://doi.org/10.1083/jcb.201912159

Gavard, J., Patel, V., Gutkind, J.S., 2008. Angiopoietin-1 prevents VEGF-induced endothelial permeability by      sequestering Src through mDia. Dev. Cell 14, 25–36. https://doi.org/10.1016/j.devcel.2007.10.019

Gocheva, V., Wang, H.W., Gadea, B.B., Shree, T., Hunter, K.E., Garfall, A.L., Berman, T., Joyce, J.A., 2010. IL-4 induces cathepsin protease activity in tumor-associated macrophages to promote cancer growth and invasion. Genes Dev. 24, 241–255. https://doi.org/10.1101/gad.1874010

Goumans, M.-J., ten Dijke, P., 2018. TGF-β signaling in control of cardiovascular function. Cold Spring Harb. Perspect. Biol. 10, a022210. https://doi.org/10.1101/cshperspect.a022210

Guo, H., Ge, Y., Li, X., Yang, Y., Meng, J., Liu, J., Wang, C., Xu, H., 2017. Targeting the CXCR4/CXCL12 axis with the peptide antagonist E5 to inhibit      breast tumor progression. Signal Transduct. Target. Ther. 2, 17033. https://doi.org/10.1038/sigtrans.2017.33

Hattori, Y., Yamada, H., Mori, H., Oba, S., Yokota, K., Omi, M., Yamamoto, Y., Toyama, K., Ohnaka, M., Takahashi, K., Imai, H., 2024. The effect of fibroblast growth factor 2 on neovascular vessels depends on      the stage of angiogenesis. Heliyon 10, e39843. https://doi.org/10.1016/j.heliyon.2024.e39843

Hicklin, D.J., Ellis, L.M., 2005. Role of the vascular endothelial growth factor pathway in tumor growth and      angiogenesis. J. Clin. Oncol. 23, 1011–1027. https://doi.org/10.1200/JCO.2005.06.081

Huang, R., Andersen, L.M.K., Rofstad, E.K., 2017. Metastatic pathway and the microvascular and physicochemical      microenvironments of human melanoma xenografts. J. Transl. Med. 15, 203. https://doi.org/10.1186/s12967-017-1307-4

Jiang, Y., Huang, J., Huang, Z., Li, W., Tan, R., Li, T., Chen, Z., Tang, X., Zhao, Y., Qiu, J., Li, C., Chen, H., Yang, Z., 2023. ADAMTS12 promotes oxaliplatin chemoresistance and angiogenesis in gastric      cancer through VEGF upregulation. Cell. Signal. 111, 110866. https://doi.org/10.1016/j.cellsig.2023.110866

Kaplan, R.N., Riba, R.D., Zacharoulis, S., Bramley, A.H., Vincent, L., Costa, C., MacDonald, D.D., Jin, D.K., Shido, K., Kerns, S.A., Zhu, Z., Hicklin, D., Wu, Y., Port, J.L., Altorki, N., Port, E.R., Ruggero, D., Shmelkov, S. V, Jensen, K.K., Rafii, S., Lyden, D., 2005. VEGFR1-positive haematopoietic bone marrow progenitors initiate the      pre-metastatic niche. Nature 438, 820–827. https://doi.org/10.1038/nature04186

Kikuchi, R., Nakamura, K., MacLauchlan, S., Ngo, D.T.-M., Shimizu, I., Fuster, J.J., Katanasaka, Y., Yoshida, S., Qiu, Y., Yamaguchi, T.P., Matsushita, T., Murohara, T., Gokce, N., Bates, D.O., Hamburg, N.M., Walsh, K., 2014. An antiangiogenic isoform of VEGF-A contributes to impaired      vascularization in peripheral artery disease. Nat. Med. 20, 1464–1471. https://doi.org/10.1038/nm.3703

Kujawski, M., Kortylewski, M., Lee, H., Herrmann, A., Kay, H., Yu, H., 2008. Stat3 mediates myeloid cell-dependent tumor angiogenesis in mice. Journal of Clinical Investigation 118, 3367–3377. https://doi.org/10.1172/JCI35213

Lee, Y.-J., Lee, H.-J., Choi, S.-H., Jin, Y.B., An, H.J., Kang, J.-H., Yoon, S.S., Lee, Y.-S., 2012. Soluble HSPB1 regulates VEGF-mediated angiogenesis through their direct      interaction. Angiogenesis 15, 229–242. https://doi.org/10.1007/s10456-012-9255-3

Li, X., Kong, X., Wang, Y., Yang, Q., 2013. 53BP1 is a novel regulator of angiogenesis in breast cancer. Cancer Sci. 104, 1420–1426. https://doi.org/10.1111/cas.12247

Lu, X., Kang, Y., 2009. Chemokine (C-C Motif) ligand 2 engages CCR2+ stromal cells of monocytic origin to promote breast cancer metastasis to lung and bone. Journal of Biological Chemistry 284, 29087–29096. https://doi.org/10.1074/jbc.M109.035899

Maisonpierre, P.C., Suri, C., Jones, P.F., Bartunkova, S., Wiegand, S.J., Radziejewski, C., Compton, D., McClain, J., Aldrich, T.H., Papadopoulos, N., Daly, T.J., Davis, S., Sato, T.N., Yancopoulos, G.D., 1997. Angiopoietin-2, a natural antagonist for Tie2 that disrupts in vivo      angiogenesis. Science 277, 55–60. https://doi.org/10.1126/science.277.5322.55

Moore, M.C., Pandolfi, V., McFetridge, P.S., 2015. Novel human-derived extracellular matrix induces in vitro and in vivo      vascularization and inhibits fibrosis. Biomaterials 49, 37–46. https://doi.org/10.1016/j.biomaterials.2015.01.022

Noh, K., Bach, D.-H., Choi, H.-J., Kim, M.S., Wu, S.Y., Pradeep, S., Ivan, C., Cho, M.-S., Bayraktar, E., Rodriguez-Aguayo, C., Dasari, S.K., Stur, E., Mangala, L.S., Lopez-Berestein, G., Sood, A.K., 2021. The hidden role of paxillin: localization to nucleus promotes tumor      angiogenesis. Oncogene 40, 384–395. https://doi.org/10.1038/s41388-020-01517-3

Olsson, A.-K., Dimberg, A., Kreuger, J., Claesson-Welsh, L., 2006. VEGF receptor signalling - in control of vascular function. Nat. Rev. Mol. Cell Biol. 7, 359–371. https://doi.org/10.1038/nrm1911

Padwal, M., Liu, L., Margetts, P.J., 2020. The role of WNT5A and Ror2 in peritoneal membrane injury. J. Cell. Mol. Med. 24, 3481–3491. https://doi.org/10.1111/jcmm.15034

Pak, B., Kim, M., Han, O., Lee, H.-W., Dubrac, A., Choi, W., Yang, J.M., Boyé, K., Cho, H., Citrin, K.M., Kim, I., Eichmann, A., Bautch, V.L., Jin, S.-W., 2024. ACVR1/ALK2-p21 signaling axis modulates proliferation of the venous      endothelium in the retinal vasculature. Angiogenesis 27, 765–777. https://doi.org/10.1007/s10456-024-09936-6

Raica, M., Cimpean, A.M., 2010. Platelet-derived growth factor (PDGF)/PDGF receptors (PDGFR) axis as      target for antitumor and antiangiogenic therapy. Pharmaceuticals (Basel) 3, 572–599. https://doi.org/10.3390/ph3030572

Shan, N., Zhang, X., Xiao, X., Zhang, H., Chen, Y., Luo, X., Liu, X., Zhuang, B., Peng, W., Qi, H., 2015. The role of laminin α4 in human umbilical vein endothelial cells and      pathological mechanism of preeclampsia. Reprod. Sci. 22, 969–979. https://doi.org/10.1177/1933719115570913

Shao, Y., Sun, L., Ma, B., Jin, R., Ban, Y., Li, R., Wang, J., Lian, H., Yue, H., 2023. VCAM-1 promotes angiogenesis of bone marrow mesenchymal stem cells derived      from patients with trauma-induced osteonecrosis of the femoral head by      regulating the Apelin/CCN2 pathway. Stem Cells Int. 2023, 6684617. https://doi.org/10.1155/2023/6684617

Sheibani, N., Sang, Y., Wang, S., Sorenson, C.M., 2024. Bax expression impacts postnatal retinal vascular development and      hyperoxia sensitivity. Exp. Eye Res. 248, 110107. https://doi.org/10.1016/j.exer.2024.110107

Stamatovic, S.M., Keep, R.F., Mostarica-Stojkovic, M., Andjelkovic, A. V., 2006. CCL2 Regulates Angiogenesis via Activation of Ets-1 Transcription Factor. The Journal of Immunology 177, 2651–2661. https://doi.org/10.4049/jimmunol.177.4.2651

Stetler-Stevenson, W.G., 1999. Matrix metalloproteinases in angiogenesis: a moving target for therapeutic      intervention. J. Clin. Invest. 103, 1237–1241. https://doi.org/10.1172/JCI6870

Takeda, N., Maemura, K., Imai, Y., Harada, T., Kawanami, D., Nojiri, T., Manabe, I., Nagai, R., 2004. Endothelial PAS domain protein 1 gene promotes angiogenesis through the      transactivation of both vascular endothelial growth factor and its      receptor, Flt-1. Circ. Res. 95, 146–153. https://doi.org/10.1161/01.RES.0000134920.10128.b4

Teicher, B.A., Fricker, S.P., 2010. CXCL12 (SDF-1)/CXCR4 pathway in cancer. Clin. Cancer Res. 16, 2927–2931. https://doi.org/10.1158/1078-0432.CCR-09-2329

Tian, D.-S., Qin, C., Zhou, L.-Q., Yang, S., Chen, M., Xiao, J., Shang, K., Bosco, D.B., Wu, L.-J., Wang, W., 2022. FSAP aggravated endothelial dysfunction and neurological deficits in acute      ischemic stroke due to large vessel occlusion. Signal Transduct. Target. Ther. 7, 6. https://doi.org/10.1038/s41392-021-00802-1

Volpert, O. V, Pili, R., Sikder, H.A., Nelius, T., Zaichuk, T., Morris, C., Shiflett, C.B., Devlin, M.K., Conant, K., Alani, R.M., 2002. Id1 regulates angiogenesis through transcriptional repression of      thrombospondin-1. Cancer Cell 2, 473–483. https://doi.org/10.1016/s1535-6108(02)00209-x

Wang, B., Sun, J., Kitamoto, S., Yang, M., Grubb, A., Chapman, H.A., Kalluri, R., Shi, G.P., 2006. Cathepsin S controls angiogenesis and tumor growth via matrix-derived angiogenic factors. Journal of Biological Chemistry 281, 6020–6029. https://doi.org/10.1074/jbc.M509134200

Wang, X., Qiu, Z., Dong, W., Yang, Z., Wang, J., Xu, H., Sun, T., Huang, Z., Jin, J., 2022a. S1PR1 induces metabolic reprogramming of ceramide in vascular endothelial      cells, affecting hepatocellular carcinoma angiogenesis and progression. Cell Death Dis. 13, 768. https://doi.org/10.1038/s41419-022-05210-z

Wang, X., Qiu, Z., Dong, W., Yang, Z., Wang, J., Xu, H., Sun, T., Huang, Z., Jin, J., 2022b. S1PR1 induces metabolic reprogramming of ceramide in vascular endothelial      cells, affecting hepatocellular carcinoma angiogenesis and progression. Cell Death Dis. 13, 768. https://doi.org/10.1038/s41419-022-05210-z

Wang, Y., Li, Z., Xu, P., Huang, L., Tong, J., Huang, H., Meng, A., 2011. Angiomotin-like2 gene (amotl2) is required for migration and proliferation      of endothelial cells during angiogenesis. J. Biol. Chem. 286, 41095–41104. https://doi.org/10.1074/jbc.M111.296806

Wen, Y., Zhou, X., Lu, M., He, M., Tian, Y., Liu, L., Wang, M., Tan, W., Deng, Y., Yang, X., Mayer, M.P., Zou, F., Chen, X., 2019. Bclaf1 promotes angiogenesis by regulating HIF-1α transcription in      hepatocellular carcinoma. Oncogene 38, 1845–1859. https://doi.org/10.1038/s41388-018-0552-1

Wu, Y., Zheng, Y., Shen, Z., Ge, W., Xie, Y., Li, C., 2014. Endostar combined with radiotherapy increases radiation sensitivity by      decreasing the expression of TGF-β1, HIF-1α and bFGF. Exp. Ther. Med. 7, 911–916. https://doi.org/10.3892/etm.2014.1526

Yang, Y., Xin, X., Fu, X., Xu, D., 2018. Expression pattern of human SERPINE2 in a variety of human tumors. Oncol. Lett. https://doi.org/10.3892/ol.2018.7819

Yao, X.-X., Lu, J.-B., Ye, Z.-D., Zheng, L., Wang, Q., Lin, Z.-Q., Liu, H., Wan, H., Fu, F.-Y., Huang, X.-Y., Xiu, J.-C., Liu, Z.-J., Hu, Y.-W., 2017. Hairy/enhancer of split homologue-1 suppresses vascular endothelial growth      factor-induced angiogenesis via downregulation of osteopontin expression. Sci. Rep. 7. https://doi.org/10.1038/s41598-017-01018-6

Yasuda, M., Shimizu, S., Ohhinata, K., Naito, S., Tokuyama, S., Mori, Y., Kiuchi, Y., Yamamoto, T., Shimizu, S., Ohhi-, K., Naito, S., Tokuyama, S., Mori, Y., Yamamoto, T., 2025. Differential roles of ICAM-1 and E-selectin in polymorphonuclear leukocyte-induced angiogenesis 1–5.

Zhao, Y., Hu, J., Sun, X., Yang, K., Yang, L., Kong, L., Zhang, B., Li, F., Li, C., Shi, B., Hu, K., Sun, A., Ge, J., 2021. Loss of m6A demethylase ALKBH5 promotes post-ischemic angiogenesis via      post-transcriptional stabilization of WNT5A. Clin. Transl. Med. 11, e402. https://doi.org/10.1002/ctm2.402
